# Beyond binary: a novel anatomical-geographical-biological framework for perineural invasion risk stratification in oral squamous cell carcinoma – a proof-of-concept study

**DOI:** 10.3389/fonc.2026.1897905

**Published:** 2026-10-12

**Authors:** Shanthi Velusamy, Prajwal Dange, Karthik N. Rao, Teertha Shetty, Nethra R., Rekha V. Kumar, Karthik S. Rishi, Aruna Korlimarla, Srinath B.S., Sreeram M.P.

**Affiliations:** 1 Department of Histopathology, Sri Shankara Cancer Hospital and Research Centre, Bengaluru, India; 2 Department of Head and Neck Surgical Oncology, Sri Shankara Cancer Hospital and Research Centre, Bengaluru, India; 3 Department of Radiation Oncology, Sri Shankara Cancer Hospital and Research Centre, Bengaluru, India; 4 Department of Molecular Oncology, Sri Shankara National Centre for Cancer Prevention and Research, Sri Shankara Cancer Foundation, Bengaluru, India; 5 Department of Surgical Oncology, Sri Shankara Cancer Hospital and Research Centre, Bengaluru, India

**Keywords:** adjuvant radiotherapy, digital pathology, histopathology, oral squamous cell carcinoma, perineural invasion, prognostic score

## Abstract

**Background:**

Perineural invasion (PNI) is an adverse pathological feature in oral squamous cell carcinoma (OSCC) and an indication for adjuvant radiotherapy but is conventionally reported as a binary variable. The prognostic relevance of its quantitative morphological features remains incompletely defined.

**Materials and methods:**

In this single-centre retrospective cohort, 101 consecutive patients with pathologically node-negative OSCC treated by curative-intent resection (January 2023 – March 2024) were analysed. Six block-level morphological PNI parameters were assessed on digitised H&E whole-slide images by two pathologists: largest involved nerve diameter (P1), maximum nerves involved (P2), pattern of invasion (P3; intratumoural, extratumoural or both), maximum circumferential extent (P4), nerves with mitotic figures (P5), and minimum distance from the tumour front to the nearest uninvolved nerve (P6). Interobserver reproducibility was evaluated for all five PNI parameters. Cutpoints were derived by maximally selected rank statistics. Univariate and bivariable Cox regression, bootstrap stability, and a composite 0–6 score were evaluated against disease-free survival (DFS) and locoregional control (LRC).

**Results:**

PNI was identified in 34 of 100 evaluable patients; binary comparison showed a non-significant trend toward inferior DFS (p = 0.104) and no LRC difference (p = 0.490). Among 30 of the 34 PNI-positive patients with complete morphological data (11 DFS events; median follow-up 20.7 months), nerve diameter >429 µm was the strongest predictor of DFS (HR 3.96, 95% CI 1.18–13.02, p = 0.026), and the most consistently selected variable in LASSO bootstrap resampling (64.8%). Large nerve calibre (P1), an extratumoral component (P3) and intraneural mitoses (P5) co-occurred as an aggressive triad (pairwise φ 0.52–0.61). The composite 0–6 score was associated with DFS (HR 1.34 per point, 95% CI 1.01–1.77, p = 0.044) and stratified patients into low-risk (0–2) and high-risk (3–6) groups for DFS (p = 0.003) and LRC (p = 0.045).

**Conclusions:**

PNI in node-negative OSCC is biologically significant and prognostically influential even within this favourable-prognosis (pN0, R0) subgroup; quantitative morphological characterisation, particularly of invaded nerve diameter, adds prognostic information beyond binary reporting. A composite morphological score may identify PNI-positive patients for future prospective evaluation of risk-adapted adjuvant strategies; external multicentre validation is required before clinical application.

## Introduction

Oral squamous cell carcinoma (OSCC), a major subset of head and neck cancers, contributes approximately 2–3% of the global cancer burden, with an increasing incidence in many regions, largely attributable to tobacco use, smoking, alcohol consumption, and related risk factors (1). Patients with OSCC present at varying stages of disease, and treatment modalities include surgery, radiotherapy, chemotherapy, and immunotherapy. Treatment decisions are guided by multiple clinicopathological parameters, including tumour–node–metastasis (TNM) staging, margin status, depth of invasion (DOI), worst pattern of invasion (WPOI), lymphovascular invasion (LVI), and perineural invasion (PNI). In early node-negative OSCC, these parameters play a particularly critical role in determining the need for adjuvant therapy, with special emphasis on DOI, WPOI, PNI, LVI, and margin status (2).

Among these factors, PNI has been consistently validated as an independent adverse prognostic indicator and is widely considered an indication for postoperative radiotherapy (3). The assessment of PNI is conventionally performed by pathologists using light microscopy.

The definition of PNI varies in the literature and includes tumour infiltration within the peripheral nerve sheath with involvement of the epineurium, perineurium, or endoneurium (Liebig type A), and/or tumour involving at least 33% of the nerve circumference (Liebig type B) (4). The detection of PNI is influenced by the definition employed, adequacy of sampling, and meticulous histopathological examination (5). In routine practice, PNI is typically reported as present or absent and may be further categorized as intra- or extratumoural.

The extent and location of PNI are features of potential prognostic importance, yet these parameters are not currently mandatory reporting elements in the College of American Pathologists (CAP) *Protocol for the Examination of Specimens from Patients with Cancers of the Lip and Oral Cavity* (version 4.2.0.0, 2023), and consensus calls for their incorporation as core data elements have so far not been uniformly adopted across reporting frameworks.

Despite the established prognostic significance of PNI in OSCC, relatively few studies have comprehensively evaluated detailed histomorphological parameters such as the extent of nerve involvement, number of nerves involved, nerve size, intra- versus extratumoural location, and the biological characteristics of tumour cells within the perineural niche (6). Consequently, the prognostic relevance of these features remains incompletely understood, with existing studies yielding limited and sometimes conflicting results.

This study aims to systematically analyse histomorphological variables associated with perineural invasion in OSCC and to evaluate their correlation with clinical outcomes. Notably, this analysis was performed using a digital pathology platform and, to the best of our knowledge, represents the first study to assess the mitotic activity of tumour cells within perineural invasion.

## Materials and methods

### Study design and patient selection

This single-centre retrospective cohort study was designed and reported in accordance with the Strengthening the Reporting of Observational Studies in Epidemiology (STROBE) statement for observational studies and the REporting recommendations for tumour MARKer prognostic studies (REMARK) guidelines for prognostic biomarker reporting. Institutional Ethics Committee (IEC) approval was obtained prior to study commencement (IEC reference number: SSCHRC/IEC26/221), and the requirement for individual informed consent was waived in view of the retrospective design and de-identification of patient data.

All consecutive adult patients with newly diagnosed, treatment-naïve, histopathologically confirmed oral squamous cell carcinoma (OSCC) who underwent curative-intent surgical resection at our institute between January 2023 and March 2024 were screened for inclusion. Eligibility criteria followed standard practice for OSCC prognostic studies and the National Comprehensive Cancer Network (NCCN) Head and Neck Cancers guidelines: (i) age 18 years or older; (ii) histopathologically confirmed primary OSCC of the oral cavity (buccal mucosa, floor of mouth, retromolar trigone, gingivo-buccal sulcus, hard palate, lip, or tongue); (iii) primary curative-intent en bloc surgical resection with concurrent ipsilateral or bilateral elective selective neck dissection of levels I–III (extending to level IV for tongue primaries) in clinically and radiologically node-negative necks; (iv) pathologically node-negative (pN0) disease confirmed on final histopathology with an adequate nodal yield (minimum 15 lymph nodes per neck) in keeping with International Collaboration on Cancer Reporting (ICCR) recommendations; (v) microscopically negative surgical margins (R0 resection, defined as a clear margin of ≥5 mm); and (vi) availability of complete archival haematoxylin and eosin (H&E)-stained pathology blocks for systematic re-review. Exclusion criteria were distant metastasis at presentation, recurrent or persistent OSCC at study entry, prior chemotherapy or radiotherapy for any malignancy, synchronous head and neck primary tumour, microscopically positive (R1) or grossly positive (R2) surgical margins, occult nodal disease identified on subsequent re-review, and loss to follow-up before the first scheduled postoperative review. Patients were followed up at the study institution; salvage management at recurrence was directed by the institutional multidisciplinary tumour board, and a subset of patients received subsequent salvage treatment at external institutions according to patient preference and logistic considerations.

### Clinicopathological data and endpoints

Clinicopathological data were extracted from institutional clinical and pathology databases. All resection specimens had been reported per the College of American Pathologists (CAP) protocol for cancers of the lip and oral cavity (version 4.2.0.0, 2023). Variables recorded included age, sex, primary subsite, pathological T stage, pathological N stage and nodal yield, surgical margin status, depth of invasion (DOI, in mm), lymphovascular invasion (LVI; present/absent), worst pattern of invasion (WPOI; categorised as 1–4 versus 5), and administration and type of adjuvant therapy. The baseline demographic and clinicopathological characteristics of the analytic cohort, stratified by perineural invasion status, are presented in **Table 1**.

**Table 1:** Demographic and clinicopathological characteristics of the study cohort, stratified by PNI status (n = 100).

Characteristic	Total (n = 100)	PNI absent (n = 66)	PNI present (n = 34)	p value
T stage				<0.001
T1	22 (22.0)	21 (31.8)	1 (2.9)	
T2	32 (32.0)	24 (36.4)	8 (23.5)	
T3	25 (25.0)	8 (12.1)	17 (50.0)	
T4a	20 (20.0)	12 (18.2)	8 (23.5)	
T4b	1 (1.0)	1 (1.5)	0 (0.0)	
Subsite				0.457
Buccal mucosa	47 (47.0)	34 (51.5)	13 (38.2)	
Tongue	39 (39.0)	23 (34.8)	16 (47.1)	
Other oral cavity	14 (14.0)	9 (13.6)	5 (14.7)	
DOI (mm), median [IQR]	8.0 [5.0–13.0]	6.0 [4.0–9.0]	12.0 [10.0–15.0]	<0.001
Lymphovascular invasion, present	27 (27.0)	11 (16.7)	16 (47.1)	0.002
WPOI grade 5	28 (28.0)	10 (15.2)	18 (52.9)	<0.001
Adjuvant therapy				0.206
Surgery alone	81 (81.0)	54 (81.8)	27 (79.4)	
Surgery + radiotherapy	15 (15.0)	11 (16.7)	4 (11.8)	
Surgery + chemoradiotherapy	4 (4.0)	1 (1.5)	3 (8.8)	

Values are n (%) unless otherwise indicated; p values are from Fisher exact test for categorical variables and Wilcoxon rank-sum test for depth of invasion.

Disease-free survival (DFS) was defined as the time from the date of primary surgery to the first event of locoregional recurrence, distant metastasis, or death from any cause; patients without an event were censored at the date of last follow-up. Locoregional control (LRC) was defined as the time from surgery to histologically or radiologically confirmed local or regional recurrence; patients with distant-only failure were censored at the date of distant metastasis, and patients who died without locoregional recurrence were censored at the date of death (no formal competing-risks framework was applied, in keeping with reporting practice in comparable single-institution OSCC cohorts; an interpretive caveat is noted in the Discussion).

### Adjuvant therapy protocol

Adjuvant treatment decisions were determined by the institutional head and neck multidisciplinary tumour board in accordance with the National Comprehensive Cancer Network (NCCN) Head and Neck Cancers guidelines and contemporary head and neck oncology practice. Postoperative radiotherapy (PORT) was delivered when one or more adverse pathological risk factors were present on final histopathology, including pT3 or pT4 primary tumour, perineural invasion, lymphovascular invasion, depth of invasion greater than 10 mm, worst pattern of invasion type 5, or close surgical margin (defined as a clear margin of less than 5 mm); patients with multiple concurrent risk factors were preferentially offered adjuvant therapy, and the addition of concurrent platinum-based chemotherapy was considered on an individualised basis in those with cumulative high-risk pathological features. Adjuvant radiotherapy was delivered using intensity-modulated radiotherapy (IMRT), volumetric-modulated arc therapy (VMAT) or three-dimensional conformal radiotherapy (3DCRT) to the primary tumour bed and at-risk ipsilateral or bilateral neck regions, to a total dose of 60 Gy in 30 daily fractions of 2 Gy delivered five fractions per week over six weeks, with an integrated boost to 66 Gy in 33 fractions for cases with close surgical margins. PORT was initiated within six weeks of primary surgery whenever clinically feasible, in keeping with NCCN recommendations on package time.

### Histopathological assessment of perineural invasion

Archived H&E-stained histopathology slides for each case were retrieved and subjected to whole-slide imaging on a Philips digital scanner (UFS-300, Philips Healthcare). New histological sections were prepared from the original tissue blocks for cases in which the archived slides were of inadequate quality. All slides containing tumour cells were digitised and independently reviewed by two experienced head and neck pathologists (S.V. and N.R.) who were strictly blinded to all clinical and outcome data throughout the morphological assessment; neither pathologist had access to recurrence status, survival data, or adjuvant treatment allocation at the time of slide review. Perineural invasion (PNI) was defined according to the criteria of Liebig et al. as the presence of tumour cells within any of the three layers of the nerve sheath (epineurium, perineurium, or endoneurium) or as tumour cells encircling at least 33% of the nerve circumference; involvement of less than one-third of the circumference without sheath infiltration was not considered diagnostic of PNI.

Six morphological parameters of PNI were systematically recorded for each slide (**Table 2**). Operational definitions were as follows: (i) P1, defined as the diameter of the largest involved nerve, measured in micrometres on the digital slide using the Philips annotation tools, measured between the outer borders of the perineurium at its maximum width; (ii) P2, defined as the count of distinct nerves involved by tumour, recorded per slide and aggregated to the patient as the maximum across all blocks (P2_MAX); (iii) P3, defined by the anatomical location of the involved nerve relative to the tumour mass, classified as intratumoral (IT), extratumoral (ET), or both (IT&ET); (iv) P4, defined as the circumferential extent of nerve involvement, expressed as a percentage; (v) P5, defined as the number of involved nerves containing one or more mitotic figures in the discrete tumour cells involving the nerve; cases with PNI and corresponding tumour cells contiguous indefinitely with the main tumour mass were excluded to avoid ambiguity; and (vi) P6, defined as the minimum distance in micrometres between the advancing tumour front (the deepest invasive edge of the tumour) and the nearest uninvolved nerve, measured digitally on slides without demonstrable PNI. The use of a binary threshold (present/absent) for intraneural mitotic activity (P5) was preferred over a formal mitotic count, as no standardised metric currently exists for enumerating mitotic figures within tumour cells invading a nerve; a formal count would require normalisation by the number of tumour cells per nerve cross-section and the total number of invaded nerves. The number of involved nerves showing intraneural mitotic activity may more directly reflect the overall aggressiveness of perineural tumour spread.

**Table 2:** Definition and methodology of PNI parameters recorded.

Parameter	Definition	Methodology
P1	Diameter of the largest nerve involved by tumour	All nerves involved by tumour in each slide were annotated for their maximum diameter; the largest nerve diameter was recorded.
P2	Number of nerves involved by tumour	The total number of nerves involved by tumour in each slide, irrespective of diameter or location, was recorded.
P3	Location of nerve involved: intratumoral vs extratumoral or both	The location of involved nerves, classified as intratumoral, extratumoral, or a combination of both, was recorded.
P4	Percentage of nerve circumference involved by tumour	The percentage of nerve circumference involved by tumour (only if fulfilling PNI criteria) was recorded.
P5	Number of nerves with mitotic activity in tumour cells involving nerve	The number of nerves with mitotic activity within infiltrating tumour cells in PNI foci was recorded. Areas where tumour completely encircles the nerve and is contiguous with the main tumour mass were excluded to prevent ambiguity. Values were summed across all blocks to give a patient-level count.
P6	Distance between advancing tumour front and nearest uninvolved nerve	In slides without nerve involvement, the distances between nerves close to the advancing tumour edge were annotated and the minimum measurement was recorded.

All slides were reviewed independently by both pathologists, with discordant cases discussed and reconciled on the digital images until a consensus opinion was reached; consensus values were used for all subsequent analyses. Block-level data were aggregated to the patient level as follows: P1 = maximum nerve diameter across all blocks (i.e. for each block, the largest involved nerve on that slide was identified by systematically reviewing all PNI-positive nerves; the patient-level value is the largest single-nerve diameter recorded across the entire case); P2 = maximum count of involved nerves across all blocks (i.e. the highest single-block burden, not the sum across blocks; the maximum was preferred over the sum because summing across blocks would conflate biological burden with the number of blocks submitted, which varies across cases); P3 = worst pattern across all blocks using the hierarchy intratumoural+extratumoural > extratumoural > intratumoural; P4 = maximum circumferential extent across all involved nerves; P5 = sum across all blocks of the number of nerves containing mitotic figures; and P6 = minimum distance across all blocks. P6 was measured in PNI-positive cases on slides not demonstrating PNI and in all PNI-negative cases; the parameters recorded by PNI status and slide type are summarised in **Table 3**.

**Table 3:** Parameters recorded by PNI status and slide type.

Case Level	Slide Level	Parameters recorded
PNI-positive	Slide with demonstrable PNI	P1, P2, P3, P4, P5
PNI-positive	Slide without demonstrable PNI	P6
PNI-negative	All slides with tumour cells	P6 only

Reproducibility of the morphological assessment was evaluated in all 101 patients by comparing independently recorded block-level measurements from both pathologists (S.V. and N.R.). Intraclass correlation coefficients (ICC; two-way mixed model, absolute agreement, single measures) were calculated for continuous parameters (P1, P2, and P6), and Cohen’s kappa (κ) (7) was computed for categorical parameters (P3 and P5). Patient-level values were derived by aggregating across blocks as described above.

Representative photomicrographs illustrating the operational definition of each PNI parameter on H&E-stained sections are shown in **Figure 1**.

**Figure 1:**
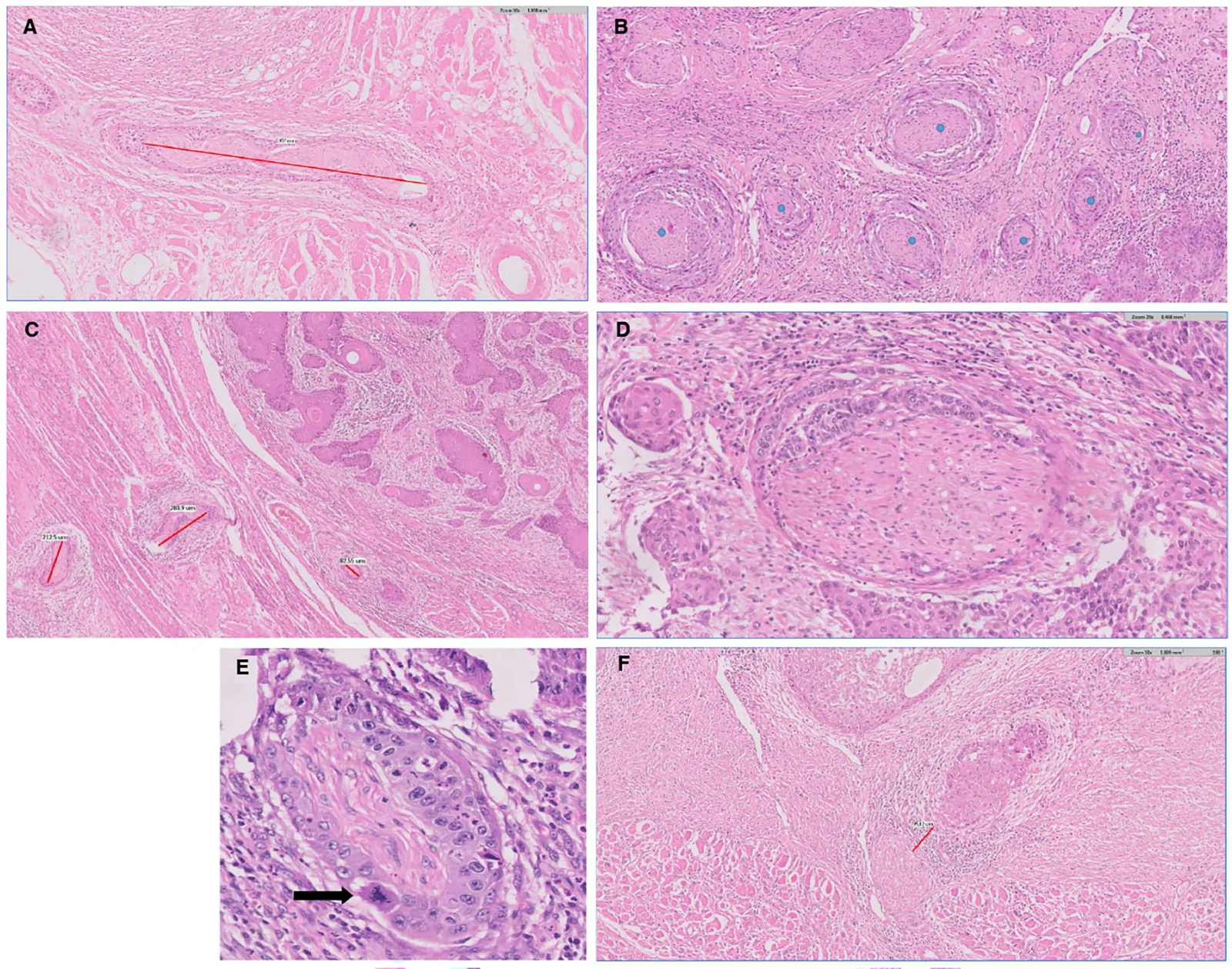
Histopathological parameters of perineural invasion (PNI) assessed on haematoxylin and eosin (H&E)-stained sections. **(A)** Measurement of the diameter of the largest nerve involved by tumour, indicated by a red line with measurement in microns (0.937 mm). (H&E stain; 10× magnification). **(B)** Count of number of nerves involved by tumour: a value of 7 nerves in this image, each indicated by blue circles. (H&E stain; 10× magnification). **(C)** Location of nerve involved (intratumoral vs extratumoral vs both): 3 extratumoral PNI foci indicated by red lines with measurements (212.5 µm, 280.9 µm and 82.55 µm) in this image. (H&E stain; 4× magnification). **(D)** Percentage of nerve circumference involved by tumour: approximately 40% circumferential involvement demonstrated in this image. (H&E stain; 20× magnification). **(E)** Number of nerves with mitotic activity in tumour cells involving the nerve: arrow indicating a mitotic image in tumour cells involving the nerve. (H&E stain; 40× magnification). **(F)** Nearest uninvolved nerve in PNI-negative cases: the red line (95.02 µm) indicates the distance to the nearest uninvolved nerve in a PNI-negative case. (H&E stain; 10× magnification).

### Statistical analysis

Continuous variables are summarised as medians and interquartile ranges (IQR) and categorical variables as counts and percentages. Survival analysis used the Kaplan–Meier method with log-rank testing for between-group comparisons. Potential follow-up was estimated using the reverse Kaplan–Meier method, in which the censoring indicator is reversed to estimate the time to censoring rather than to event. Hazard ratios (HR) with 95% confidence intervals (CI) were estimated by Cox proportional hazards regression. The proportional hazards assumption was assessed visually by log–log plots and by Schoenfeld residual tests.

Owing to the limited number of recurrence events, the analytic plan was specified up-front as exploratory and hypothesis-generating, with the events-per-variable (EPV) constraint guiding model complexity. Optimal dichotomisation cutpoints for the continuous PNI parameters (P1, P2, P5, P6) were derived from the data using maximally selected rank statistics (maxstat), implemented in the R *maxstat* package, with disease-free survival as the test outcome, the log-rank statistic as the test, the Lau (1994) approximation for the asymptotic null distribution and bias correction (the Lausen–Schumacher correction), and minprop = 0.20/maxprop = 0.80 to constrain the search to clinically meaningful group sizes. We acknowledge that maxstat-derived cutpoints inflate type I error and overstate effect size in small samples; the wide confidence intervals on individual hazard ratios should be interpreted in this light, and external validation is required before any cutpoint is treated as clinically actionable. Cutpoints were derived from the same dataset used to test their prognostic value, and we have not performed split-sample or held-out validation; the resulting C-index is therefore an in-sample optimistic estimate.

LASSO (L1-penalised) Cox regression was used as a tool for ranking variable importance and was implemented using the *glmnet* package. Eight candidate variables were considered: four PNI parameters (P1, P2, P3, P5) and four clinicopathological variables (advanced T stage [T3/T4a/T4b], DOI > 10 mm, lymphovascular invasion, and WPOI grade 5). P4 was excluded *a priori* because of saturation (see Results); P6 was analysed in a parallel model owing to missing data. The optimal penalty parameter (λ) was selected by 5-fold cross-validation on the partial likelihood deviance. Because the events-per-variable ratio in this cohort (11 DFS events/8 candidate variables ≈ 1.4) is well below conventional thresholds (≥10) for unbiased estimation, formal LASSO selection was anticipated to be unstable; we therefore used non-parametric bootstrap stability across 500 resamples as the primary measure of variable importance, defining a variable as a stable independent predictor when it was selected in ≥50% of resamples. Bivariable Cox regression with P1 and each clinicopathological confounder individually was used as a sensitivity analysis to assess whether the P1 effect was attenuated by adjustment. A parsimonious multivariable Cox model containing the bootstrap-stable variables (P1 and LVI) was fitted as a sanity check; with two covariates and 11 events the EPV ratio of 5.5 remains low and the model should be considered illustrative rather than confirmatory.

Pairwise phi coefficients with Fisher’s exact test were used to describe co-occurrence among adverse features. The composite PNI score (range 0–6) was constructed by summing the dichotomised adverse status of P1, P2, P3, P5 and P6. Each binary parameter (P1, P2, P5, P6) contributed 1 point when adverse; P3 contributed 0 points for intratumoral-only PNI, 1 point for purely extratumoral PNI, and 2 points for combined intratumoural+extratumoural PNI, on the prior biological rationale that extratumoral spread implies a larger field of nerve-tracking disease and that combined patterns subsume both phenotypes. The 2-point weighting for combined patterns is biologically motivated rather than coefficient-derived. Risk-group dichotomisation at score ≥ 3 (one-third of the maximum 6-point range) was specified prior to outcome analysis as a fixed cutpoint; the same cutpoint was applied for both DFS and LRC analyses.

Missing data were handled by complete-case analysis on the analytic subset relevant to each model: P6 was missing in 4 PNI-positive patients in whom no slide demonstrating uninvolved nerves could be retrieved (assumed missing at random conditional on observed PNI status), and these patients were excluded only from analyses that required P6. No imputation was performed. Patients with zero follow-up time (n = 1) were excluded from Cox regressions but retained in descriptive tabulations. All p-values are two-sided and a threshold of 0.05 was used to define statistical significance, with the explicit caveat that no formal correction for multiple comparisons was applied; reported p-values for the multiple PNI parameters and for maxstat cutpoint searches should therefore be interpreted as nominal rather than confirmatory. Analyses were performed in R version 4.3 (R Foundation for Statistical Computing, Vienna, Austria) using the *survival, survminer, maxstat, glmnet, dplyr* and *ggplot2* packages. Analytic code is available from the corresponding author on reasonable request.

## Results

### Cohort characteristics

Of the 101 consecutive patients screened, one was excluded because PNI status could not be determined, leaving 100 evaluable patients in the analytic cohort. PNI was identified in 34 patients (34.0%) and was absent in 66 patients (66.0%). The median potential follow-up estimated by the reverse Kaplan–Meier method was 20.7 months (95% CI 18.3–23.8 months; range 0.0–34.1 months); the number at risk for the disease-free survival endpoint was 55 at 12 months and 26 at 24 months. The cohort comprised early-stage (T1/T2) tumours in 54 patients (54.0%) and advanced-stage (T3/T4a/T4b) tumours in 46 patients (46.0%). PNI was more prevalent in advanced-stage disease (T3/T4a/T4b in 73.5% of PNI-positive patients versus 31.8% of PNI-negative patients). Buccal mucosa and tongue were the predominant subsites, accounting for 47 (47.0%) and 39 (39.0%) patients respectively. Lymphovascular invasion was present in 27 patients (27.0%), and WPOI grade 5 was observed in 28 patients (28.0%). Both LVI and WPOI grade 5 were more frequent in PNI-positive than in PNI-negative cases (both p < 0.001). Full baseline demographic and clinicopathological characteristics, stratified by PNI status, are summarised in **Table 1**.

### Impact of PNI on survival outcomes

Across the full cohort (n = 100), 23 patients (23.0%) experienced a disease-free survival event (recurrence or death) and 18 (18.0%) experienced a locoregional event during follow-up. The DFS event rate was 35.3% (12/34) in PNI-positive patients and 16.7% (11/66) in PNI-negative patients; the corresponding LRC event rates were 23.5% (8/34) and 15.2% (10/66). On Kaplan–Meier analysis, PNI-positive patients showed a non-significant trend towards inferior disease-free survival (log-rank p = 0.104) (**Figure 2A**), and no significant difference was observed for locoregional control (log-rank p = 0.490) (**Figure 2B**).

**Figure 2:**
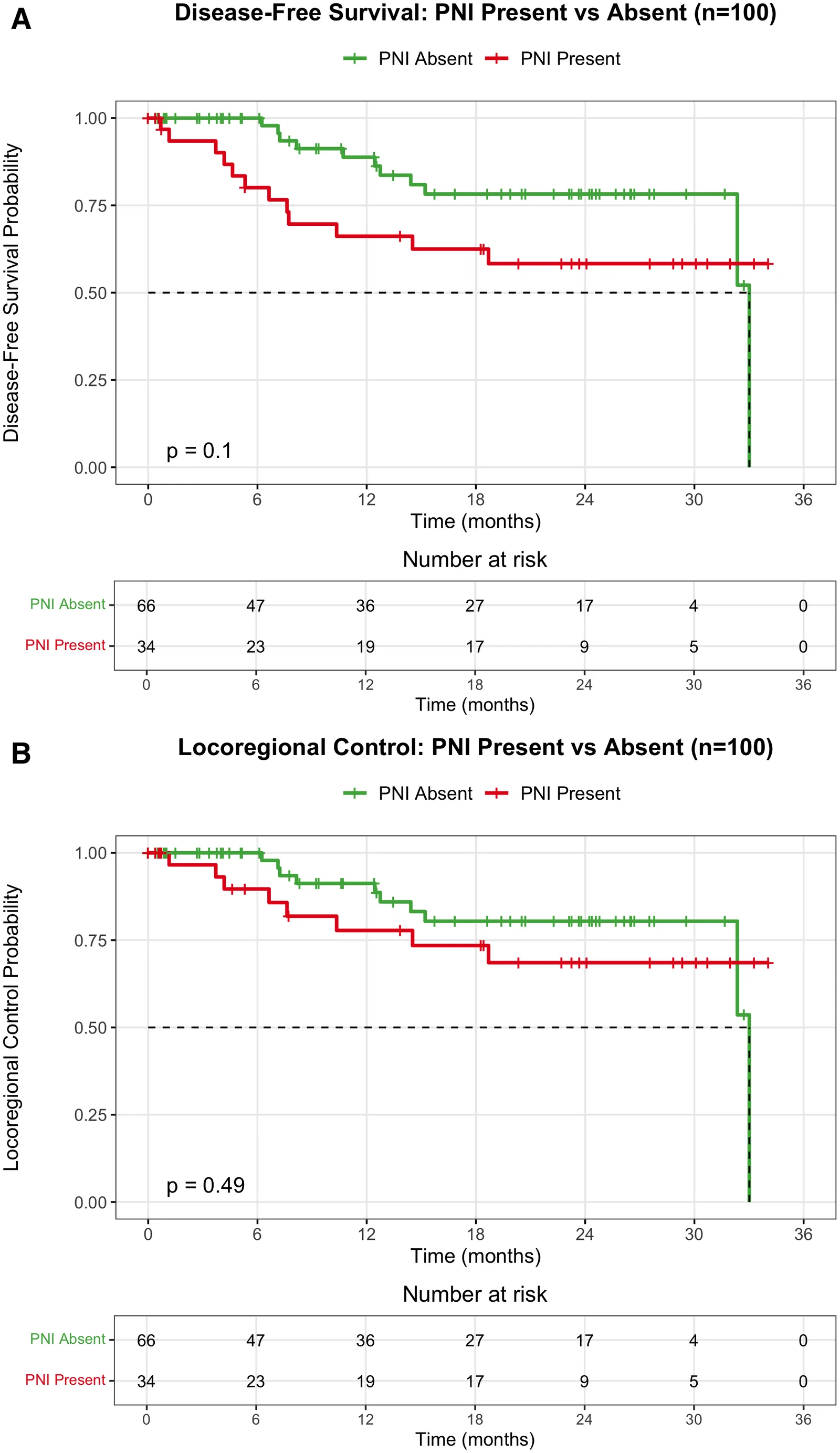
Kaplan–Meier survival curves comparing PNI-positive (n = 34) versus PNI-negative (n = 66) patients. **(A)** Disease-free survival; log-rank p = 0.104. **(B)** Locoregional control; log-rank p = 0.490. Risk tables below each curve indicate the number at risk at successive time points.

### PNI parameter characterisation

Of the 34 PNI-positive patients, 30 had complete morphological data for parameters P1–P5 and constituted the PNI parameter analytic subset; the remaining 4 patients had incomplete archival material for one or more parameters and were excluded from analyses requiring those values (**Table 3**). P6 (distance to the nearest uninvolved nerve) was measurable in 30 PNI-positive patients overall, of whom 26 also had complete P1–P5 data. Within the 30-patient subset, the largest involved nerve diameter (P1) ranged from 49.1 to 1480.0 µm (median 226.5 µm; IQR 132.1–482.8 µm). The maximum number of nerves involved in any single block (P2) ranged from 1 to 23 (median 2; IQR 1–5). The worst pattern of invasion across blocks (P3) was intratumoral only in 23 patients (76.7%) and combined intratumoral and extratumoral in 7 patients (23.3%); no patient demonstrated a purely extratumoral pattern. The maximum circumferential extent of nerve involvement (P4) was 100% in 26 patients (86.7%); the remaining 4 patients (13.3%) had partial circumferential involvement (range 5–95%). The number of nerves with mitotic activity (P5) ranged from 0 to 10 (median 0; IQR 0–1), with 12 patients (40.0%) demonstrating at least one nerve with mitoses. Within the 26-patient subset with complete data, the minimum distance to the nearest uninvolved nerve (P6) ranged from 2.6 to 838.0 µm (median 112.2 µm; IQR 59.2–206.9 µm). Maxstat-derived dichotomisation cutpoints, applied subsequently in regression and scoring analyses, were P1 > 429 µm, P2 > 1, P5 > 0 nerves, and P6 ≤ 95 µm.

### Reproducibility of morphological assessment

Interobserver agreement was excellent across all five PNI parameters (**Table 4**). For continuous parameters, ICC ranged from 0.804 (P1: largest nerve diameter; 95% CI 0.645–0.895; n = 38 cases in the interobserver re-review dataset, which includes all PNI-positive cases from the full 101-patient re-review) to 0.998 (P2: number of nerves involved; 95% CI 0.995–0.999). P6 achieved an ICC of 0.896 (95% CI 0.838–0.933; n = 96 patients). Categorical agreement was perfect for both P3 (invasive pattern, κ = 1.00) and P5 (intraneural mitotic activity, κ = 1.00). Bland–Altman analysis of the two continuous parameters most susceptible to digital measurement variability, P1 (bias = 84.5 µm; 95% limits of agreement −385 to +554 µm) and P6 (bias = −51.4 µm; 95% limits of agreement −355 to +253 µm), confirmed the absence of systematic proportional bias and was consistent with the anticipated 30–40% measurement variation for digitally measured nerve dimensions (**Supplementary Figures S9**, **S10**). The near-perfect agreement for categorical parameters (P3 and P5; κ = 1.000) and for P2 (ICC = 0.998) should be interpreted with caution given the small number of PNI-positive cases available for comparison (n = 38 for P1–P5 parameters, from the interobserver re-review dataset); a larger cohort would be required to establish the true extent of interobserver variation for these parameters. For P1 (largest nerve diameter), subtle differences in the angle and direction of the digital annotation line are the principal source of measurement variability: placing the caliper across a slightly different axis of the same nerve cross-section yields a different value even when both observers annotate the same nerve (**Supplementary Figure S11**). For P6 (minimum tumour-to-nerve distance), variability arises primarily from the selection of the specific site along the advancing tumour front: when the infiltrating edge spans a wide field, different observers may choose different reference points, yielding substantially different values (**Supplementary Figures S12**, **S13**). In this study, the most-advancing edge was used as the reference point, and consensus values were established following joint review of each observer’s independently chosen annotation positions.

**Table 4:** Interobserver agreement for PNI morphological parameters (n = 101 patients).

Parameter	n	Method	Statistic (95% CI)	p-value
P1: Largest nerve diameter (µm)	38	ICC	0.804 (0.645–0.895)	<0.001
P2: No. of nerves involved	38	ICC	0.998 (0.995–0.999)	<0.001
P3: Invasive pattern (IT/ET/IT&ET)	38	κ	1.000 (1.000–1.000)	<0.001
P5: Intraneural mitoses (Y/N)	38	κ	1.000 (1.000–1.000)	<0.001
P6: Tumour-nerve distance (µm)	96	ICC	0.896 (0.838–0.933)	<0.001

ICC, intraclass correlation coefficient (two-way mixed model, absolute agreement, single measures). κ: Cohen’s kappa. CI, confidence interval. P1–P5 assessed in PNI-positive patients only; P6 assessed in all patients.

### Univariate survival analysis of individual PNI parameters

On univariate Cox regression for disease-free survival (**Table 5**) within the 30-patient scoring subset (11 DFS events), the largest involved nerve diameter (P1 > 429 µm) was associated with inferior DFS (HR 3.92, 95% CI 1.18–13.02, p = 0.026). The number of nerves with mitosis (P5 > 0) yielded HR 2.78 (95% CI 0.84–9.18, p = 0.093) and the minimum distance to uninvolved nerve (P6 ≤ 95 µm), analysed in the 26-patient subset with complete P6 data and 10 DFS events, yielded HR 3.08 (95% CI 0.87–10.86, p = 0.081). The number of nerves involved (P2 > 1) and the presence of an extratumoral component (P3) did not reach significance (HR 1.95, 95% CI 0.57–6.67, p = 0.289 and HR 1.94, 95% CI 0.57–6.64, p = 0.293, respectively). P4 was excluded from regression because 86.7% of PNI-positive patients had complete (100%) circumferential involvement, which left the variable with insufficient between-patient variability for meaningful estimation. All confidence intervals are wide, consistent with the limited number of events. Kaplan–Meier curves stratified by each PNI parameter are shown in **Figure 3**, and the corresponding forest plot in **Figure 4**.

**Table 5:** Univariate Cox regression for disease-free survival by PNI parameter (PNI-positive scoring subset, n = 30; one patient with zero follow-up contributes no information to Cox regression and is effectively excluded by the model, so the analytic sample for **Table 7** multivariable models is n = 29).

PNI Parameter	HR	95% CI	p-value
P1: Nerve diameter (>429 µm)	3.96	1.18–13.02	0.026
P2: Nerves involved (>1)	1.95	0.57–6.67	0.289
P3: Extratumoral component	1.94	0.57–6.64	0.293
P5: Nerves with mitosis (>0)	2.78	0.84–9.18	0.093
P6: Distance ≤95 µm (n=26)	3.08	0.87–10.86	0.081

P4 excluded (non-discriminatory).

**Figure 3:**
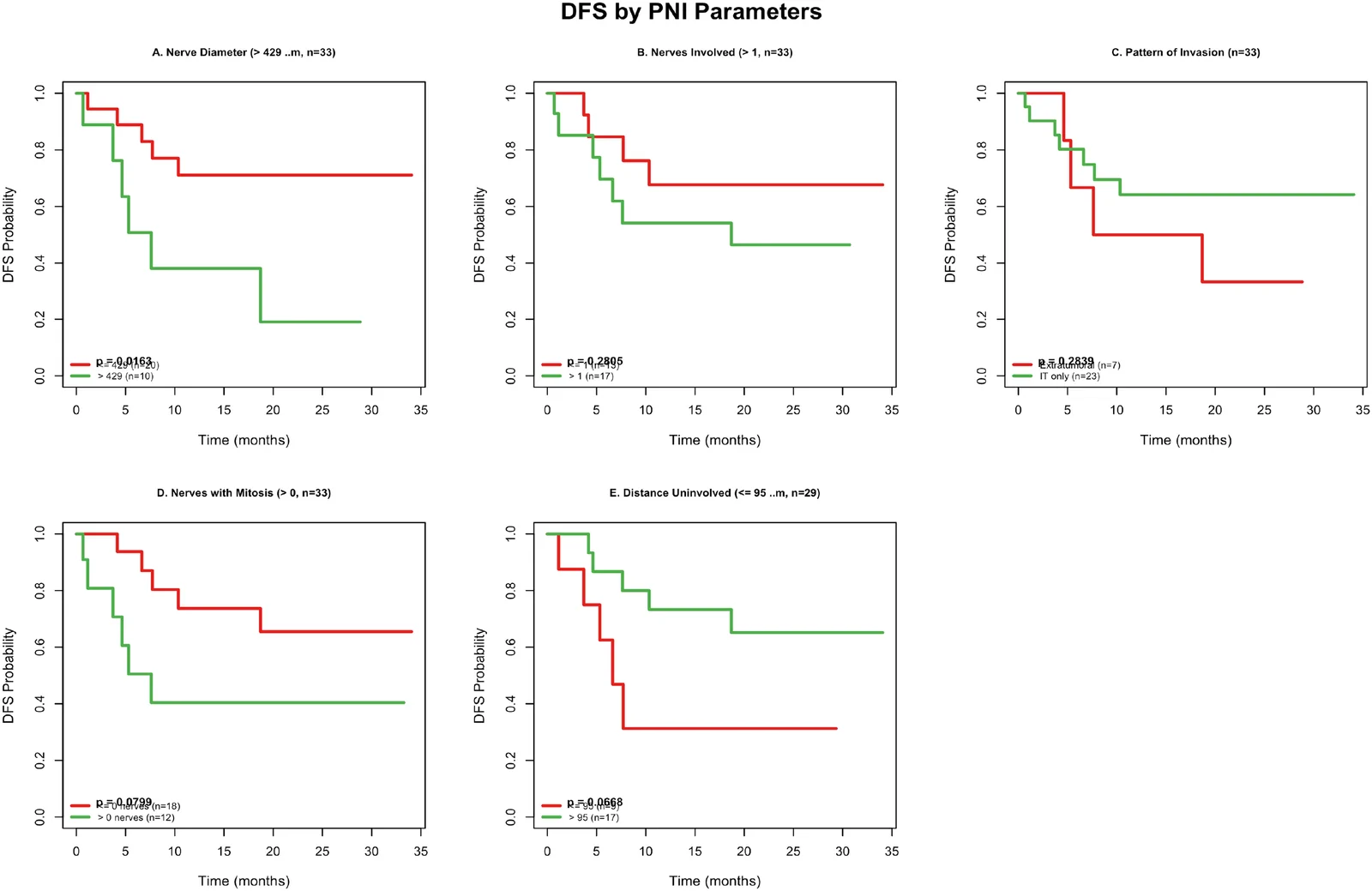
Kaplan-Meier curves for disease-free survival stratified by each PNI parameter: **(A)** nerve diameter, **(B)** number of nerves, **(C)** pattern of invasion, **(D)** nerves with mitosis, **(E)** distance to uninvolved nerve. Dashed cutpoints derived from maxstat analysis.

**Figure 4:**
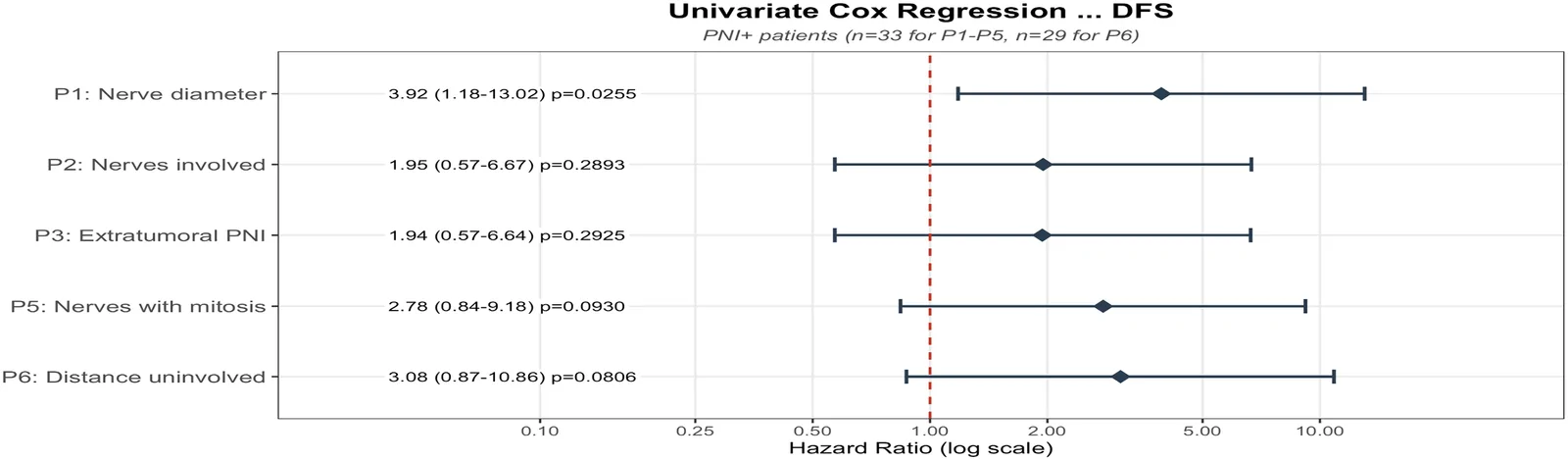
Forest plot of univariate hazard ratios for disease-free survival by PNI parameter. Diamond = point estimate; horizontal bars = 95% confidence interval; dashed red line = HR of 1.0 (no effect).

### Penalised regression and bootstrap stability

LASSO (L1-penalised) Cox regression with 5-fold cross-validation included eight candidate variables (P1, P2, P3, P5, advanced T stage, DOI > 10 mm, LVI, WPOI grade 5), with P4 excluded *a priori* for non-discrimination and P6 analysed in a separate model owing to missing data. With 11 DFS events distributed across the eight candidates (events-per-variable approximately 1.4), the cross-validation procedure selected a penalty value at which all coefficients were shrunk to zero at lambda.min, indicating that the events-per-variable ratio was insufficient for formal LASSO variable selection. Bootstrap stability analysis across 500 resamples was therefore used as the principal measure of variable importance (**Table 6**). Three variables met the ≥50% selection threshold for DFS: P1 (64.8%), LVI (55.0%), and WPOI-5 (53.2%). P5 was selected in 44.8% of resamples, P2 in 49.8%, both below the stability threshold. Advanced T stage (26.8%) and DOI > 10 mm (26.2%) did not reach the threshold. For the LRC endpoint, no variable exceeded the 50% threshold; the highest selection rates were observed for LVI (38.3%) and P1 (34.9%). LASSO coefficient paths along the penalty trajectory are shown in **Figure 5**.

**Table 6:** Bootstrap stability analysis of LASSO Cox regression (DFS).

Variable	DFS boot %	Stable?	LRC Boot %	Type
P1: Large nerve (>429 µm)	**64.8%**	**✓**	**34.9%**	**PNI**
P2: Multiple nerves (>1)	49.8%	–	17.1%	PNI
P3: Extratumoral component	29.0%	–	16.7%	PNI
P5: Nerves with mitosis (>0)	44.8%	–	30.4%	PNI
Advanced T (T3/T4a/T4b)	26.8%	–	13.3%	Clinicopath
DOI > 10 mm	26.2%	–	17.9%	Clinicopath
LVI present	**55.0%**	**✓**	**38.3%**	**Clinicopath**
WPOI = 5	**53.2%**	**✓**	**30.4%**	**Clinicopath**

Boot % = selection frequency across 500 bootstrap resamples. LRC Boot % shown for reference. Green shading = selected in ≥50% of resamples (stable predictor). Bold values indicate stable predictors (DFS bootstrap selection frequency ≥50%, the pre-specified threshold).

**Figure 5:**
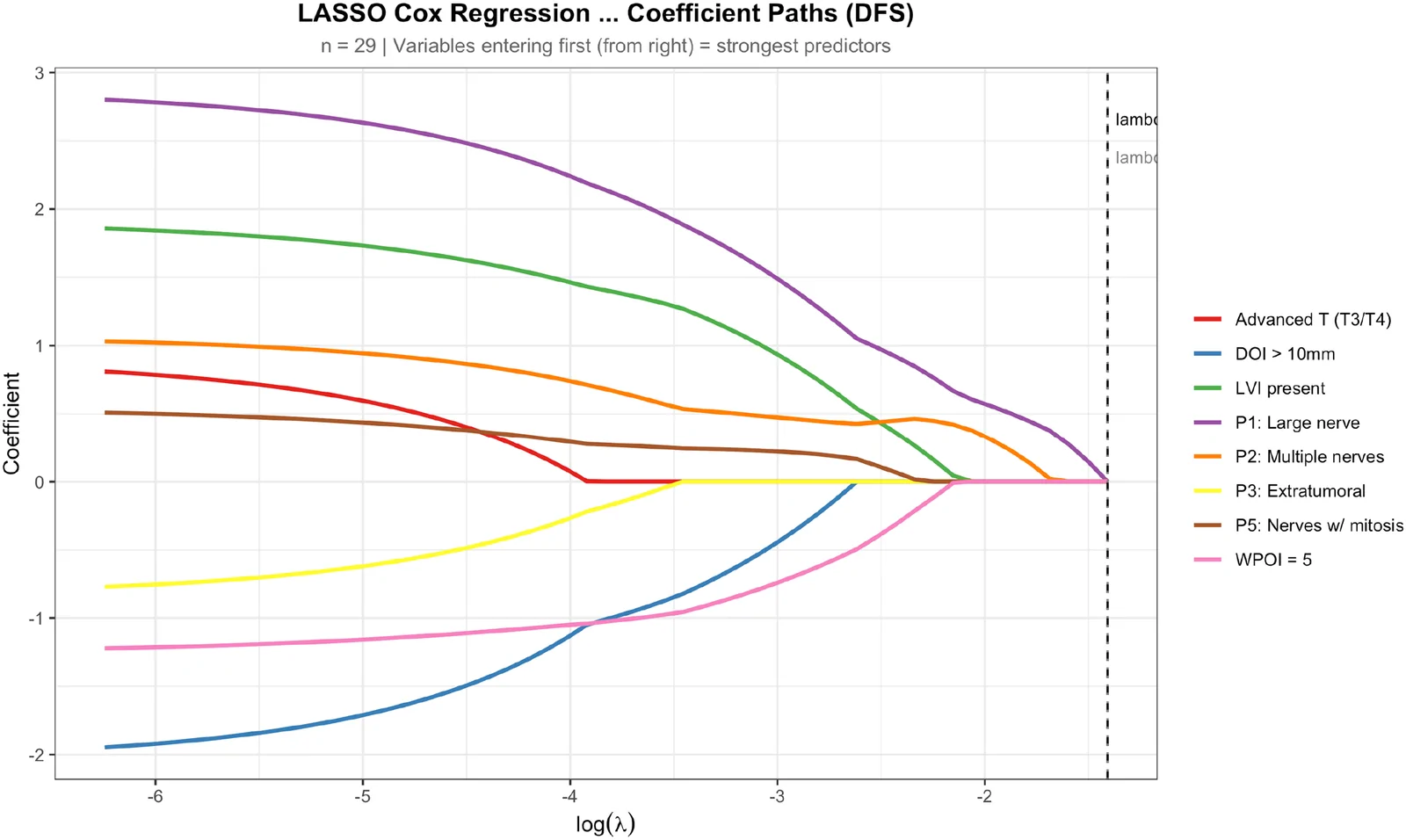
LASSO coefficient paths for disease-free survival in the PNI-positive scoring subset (n = 30; 11 DFS events). The horizontal axis represents log(lambda); each curve traces the partial-likelihood coefficient of one variable across the penalty trajectory. Vertical dashed line = lambda.min; dotted line = lambda.1se.

### Bivariable and parsimonious multivariable cox regression

Bivariable Cox regression for DFS in the PNI-positive subset with follow-up > 0 months (n = 29; 11 events) is summarised in **Table 7**. P1 (nerve diameter > 429 µm) retained statistical significance after adjustment for each clinicopathological confounder individually, as summarised in **Table 7**: unadjusted HR 3.92 (95% CI 1.18–13.02, p = 0.026), adjusted HR 4.64 (1.19–18.01, p = 0.027) for advanced T stage, 6.06 (1.21–30.40, p = 0.029) for DOI > 10 mm, 6.89 (1.68–28.31, p = 0.007) for LVI, and 4.15 (1.24–13.89, p = 0.021) for WPOI grade 5. Adjusted point estimates were higher than the unadjusted estimate; confidence intervals were wide, consistent with the limited number of events.

**Table 7:** Bivariable Cox regression: P1 (nerve diameter > 429 µm) adjusted for each confounder individually (DFS, n = 29).

Model	P1 HR	95% CI	p-value
Unadjusted	3.96	1.26–12.45	0.018
+ Advanced T (T3/T4a/T4b)	4.64	1.19–18.01	0.027
+ DOI > 10mm	6.06	1.21–30.40	0.029
+ LVI present	6.89	1.68–28.31	0.007
+ WPOI = 5	4.15	1.24–13.89	0.021

As a parsimonious sensitivity analysis informed by the bootstrap stability ranking, a multivariable Cox model containing the two bootstrap-stable variables (P1 > 429 µm and LVI present) was fitted within the same 29-patient analytic subset (events-per-variable ratio approximately 5.5). In this two-variable model, P1 retained association with DFS (HR 6.89, 95% CI 1.68–28.31, p = 0.007) and LVI showed a non-significant trend (HR 3.58, 95% CI 0.88–14.61, p = 0.076); the model concordance index was 0.72. Inclusion of WPOI grade 5 as a third covariate raised concordance to 0.79, although the WPOI coefficient in this three-variable model was negative and non-significant (HR 0.31, 95% CI 0.08–1.14, p = 0.275) and is reported as illustrative only given the very low events-per-variable ratio. The proportional hazards assumption was satisfied for all retained covariates on Schoenfeld residual testing.

### Co-occurrence of adverse PNI features

Pairwise phi coefficient analysis revealed significant clustering of adverse PNI features (**Figure 6**). The strongest pairwise associations were observed between P1 and P3 (φ = 0.61, p = 0.002), P1 and P5 (φ = 0.58, p = 0.004), and P3 and P5 (φ = 0.52, p = 0.009). P2 also co-occurred with P3 (φ = 0.48, p = 0.010) and P5 (φ = 0.44, p = 0.026). P6 (distance from tumour front to the nearest uninvolved nerve), the parameter that operationalises the nerve–tumour proximity concept proposed on the basis of spatial transcriptomic data, did not show statistically significant correlation with any other PNI parameter at the 0.05 threshold. Among clinicopathological variables, DOI and advanced T stage were highly correlated (φ = 0.67, p < 0.001); neither showed statistically significant correlation with PNI-specific parameters in the heatmap.

**Figure 6:**
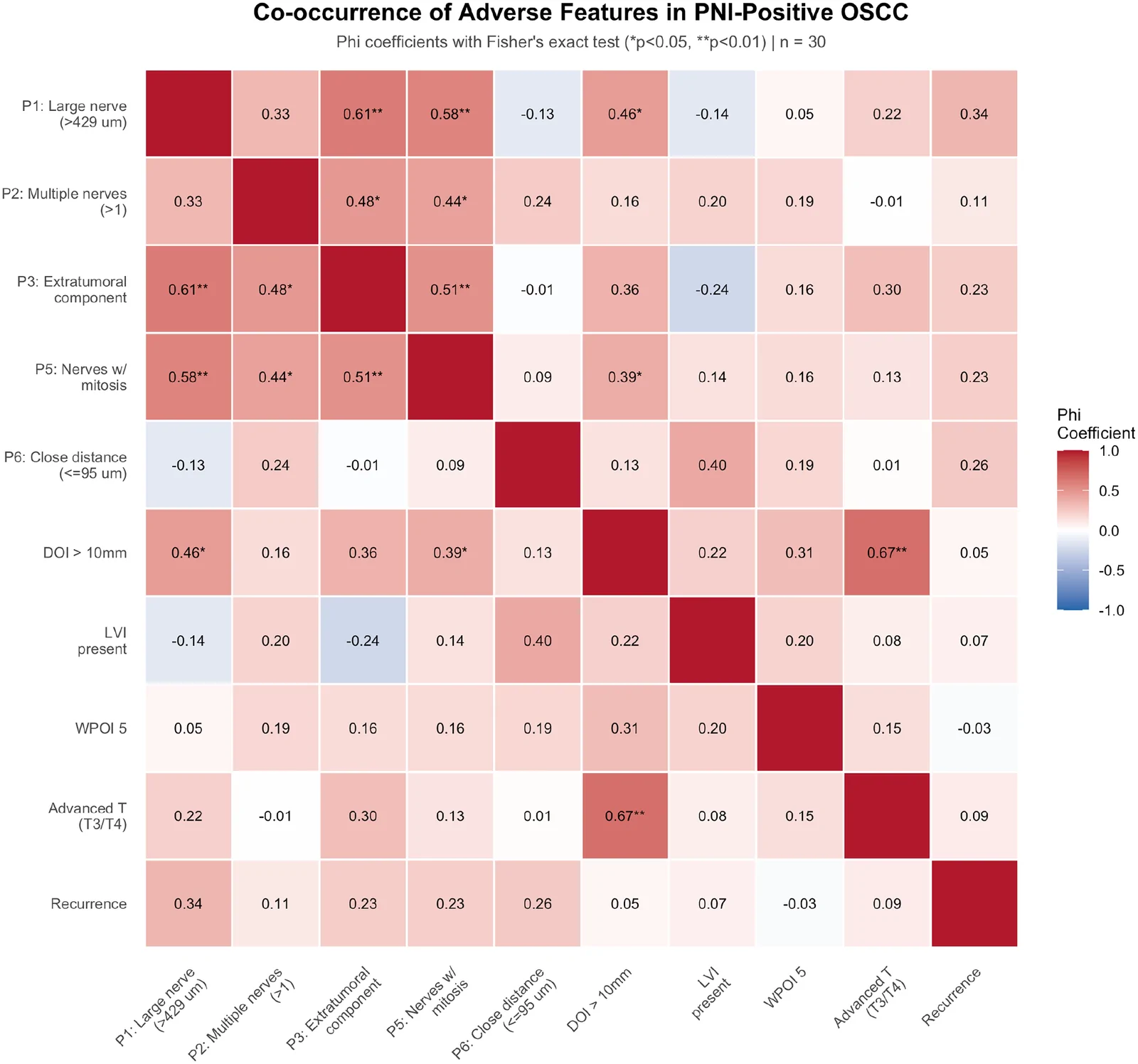
Co-occurrence heatmap of adverse features in PNI-positive OSCC (n = 30). Values represent phi coefficients; *p < 0.05, **p < 0.01 by Fisher’s exact test. Red = positive correlation; blue = negative. PNI parameters (P1, P3, P5) form a distinct cluster of co-occurring features, separate from clinicopathological variables.

### Morphological cluster

Because the three parameters P1 (large nerve calibre, >429 µm), P3 (extratumoral component) and P5 (intraneural mitotic activity) showed the strongest mutual correlation and behaved as a co-occurring cluster on the heatmap, their joint distribution was examined as a candidate aggressive PNI triad. The number of triad features present per PNI-positive patient was tabulated against DFS. Sixteen of 30 PNI-positive patients (53.3%) carried none of the three features, 6 (20.0%) carried one feature, 3 (10.0%) carried two features, and 5 (16.7%) carried the complete triad. A graded increase in DFS event rate was observed with cumulative triad burden: 25.0% in patients carrying none or one feature versus 60.0% in those carrying two or three features. When modelled as a continuous ordinal predictor in Cox regression, each additional triad feature increased the hazard of a DFS event by 63% (HR 1.63 per feature, 95% CI 1.01–2.62, p = 0.045). A direct binary comparison of complete-triad versus non-complete-triad patients showed a numerically higher DFS event rate in the complete-triad subgroup (60.0% versus 32.0%) but did not reach statistical significance (log-rank p = 0.14), reflecting the small size of the complete-triad subgroup (n = 5). These findings indicate that the three features do not occur as independent accidents but tend to co-segregate, the prognostic implications of which are further explored in the Discussion, noting that the complete-cluster subgroup was small (n = 5) and the direct binary comparison did not reach significance (log-rank p = 0.14) of which are further explored in the Discussion.

### Composite PNI score

A composite PNI score (range 0–6) was constructed by summing the binarised adverse status of five parameters: P1 (>429 µm = 1 point), P2 (>1 = 1 point), P3 (IT = 0, ET = 1, IT&ET = 2 points), P5 (>0 nerves with mitosis = 1 point), and P6 (≤95 µm = 1 point). P4 (circumferential extent) was excluded as it was non-discriminatory (86.7% of patients had 100% involvement). When analysed as a continuous predictor in Cox regression, each additional point on the PNI score was associated with a 34% increase in the hazard of recurrence (HR 1.34, 95% CI 1.01–1.77, p = 0.044; for comparison, binary PNI status in the full cohort [n = 100] yielded a concordance index of 0.636 for DFS versus 0.682 for the morphological score in the PNI-positive subset — noting these are different populations; in-sample C-index 0.682). Bootstrap optimism correction (500 resamples, cutpoints fixed at dataset-derived values) estimated a mean optimism of 0.36, yielding a corrected concordance index of 0.32; this attenuation confirms substantial in-sample overfitting given only 11 DFS events in 29 patients and reinforces that external validation is required before the score can be applied. The high-risk group (score 3–6, n = 11) showed inferior DFS compared with the low-risk group (score 0–2, n = 19; log-rank p = 0.003), with estimated 24-month DFS of 22.5% versus 75.3%; this 24-month estimate lies beyond the 95% CI of the median potential follow-up (18.3–23.8 months) and should be interpreted with caution. Locoregional control also differed between risk groups (log-rank p = 0.045). Event rates by individual PNI parameter status are shown in **Figure 7**.

**Figure 7:**
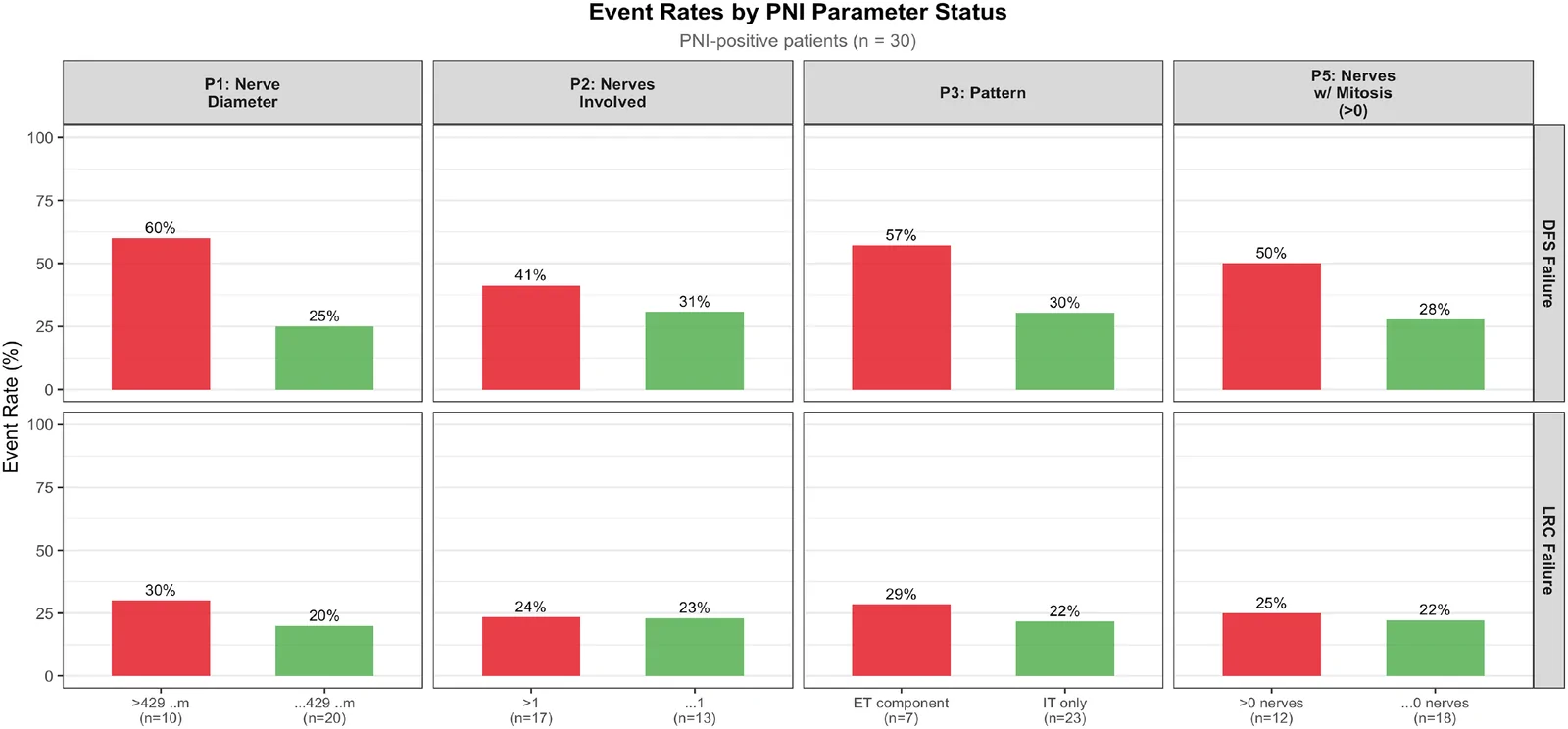
Event rates for DFS failure and LRC failure stratified by each PNI parameter status (adverse in red, favourable in green). PNI-positive scoring subset, n = 30.

### P6 analysis in PNI-negative patients

The minimum distance from tumour to the nearest uninvolved nerve (P6) was also measured in PNI-negative patients (n = 66). Using maxstat analysis, an optimal cutpoint of 72 µm was identified; however, this did not reach statistical significance for either DFS (p > 0.05) or LRC (p > 0.05). The hazard ratio for P6 ≤ 72 µm was 0.31 (95% CI 0.04–2.49, p = 0.273) for DFS, suggesting that proximity to nerves does not predict recurrence in the absence of PNI.

### Treatment failure subgroup analysis

To identify whether morphological PNI characteristics, demographic features or adjuvant therapy differed between patients who experienced treatment failure (defined as a disease-free survival event) according to PNI status, the 23 patients who experienced a DFS event during follow-up were stratified by PNI status (12 PNI-positive, 11 PNI-negative) and compared across baseline demographics, clinicopathological features, the six block-level PNI morphological parameters (P1–P6), the aggressive PNI triad, and adjuvant therapy utilisation (**Table 8**). Across all variables compared, no statistically significant difference was observed between PNI-positive and PNI-negative recurrence subgroups (all p > 0.05), reflecting the limited statistical power available within a 23-patient failure subgroup. Numerically, PNI-positive failures showed a higher prevalence of advanced T stage (75.0% versus 54.5%), lymphovascular invasion (50.0% versus 18.2%) and WPOI grade 5 (50.0% versus 27.3%), and a shorter median minimum distance from tumour front to the nearest uninvolved nerve (94.5 µm versus 211.9 µm). Within the PNI-positive failure subgroup, the morphological features of the aggressive triad were common: 50.0% had P1 > 429 µm, 58.3% had more than one nerve involved (P2 > 1), 33.3% had an intratumoral plus extratumoral pattern, 50.0% showed intraneural mitotic activity, 25.0% (3 of 12) carried the complete aggressive triad and 50.0% (6 of 12) carried two or more triad features at the time of treatment failure. Notably, although the PNI-positive failure subgroup carried a heavier adverse-feature burden, fewer of these patients had received adjuvant radiotherapy (25.0% versus 45.5%, p = 0.400) or any adjuvant therapy (25.0% versus 45.5%, p = 0.400) than the PNI-negative failure subgroup; while this difference did not reach statistical significance in this small subgroup, it is consistent with the hypothesis that under-treatment of the morphologically high-risk PNI subset contributes to the residual recurrence burden in node-negative OSCC and supports the argument for risk-stratified intensification of adjuvant therapy in patients carrying multiple adverse PNI morphological features.

**Table 8:** Clinicopathological, PNI morphological (P1–P6) and adjuvant therapy variables in PNI-positive (n = 12) versus PNI-negative (n = 11) treatment-failure patients.

Variable	PNI-positive failures (n = 12)	PNI-negative failures (n = 11)	p value
*Demographics and clinicopathological features*			
T stage			0.400
Early (T1/T2)	3 (25.0%)	5 (45.5%)	
Advanced (T3/T4a/T4b)	9 (75.0%)	6 (54.5%)	
Subsite			0.648
Buccal mucosa	4 (33.3%)	6 (54.5%)	
Tongue	6 (50.0%)	3 (27.3%)	
Other (lip/RMT)	2 (16.7%)	2 (18.2%)	
DOI (mm), median (IQR)	12.5 (6.8-13.8)	8.0 (4.2-14.0)	0.323
Lymphovascular invasion			0.193
Present	6 (50.0%)	2 (18.2%)	
Absent	6 (50.0%)	9 (81.8%)	
WPOI grade			0.400
Grade 1-4	6 (50.0%)	8 (72.7%)	
Grade 5	6 (50.0%)	3 (27.3%)	
*PNI morphological parameters (P1–P6) — PNI-positive failures only*			
P1: largest nerve diameter (µm), median (IQR)	450.0 (125.4-576.5)	n/a (PNI absent)	n/a
P1 nerve calibre			n/a
≤ 429 µm	5 (41.7%)	n/a	
> 429 µm	6 (50.0%)	n/a	
Not assessable	1 (8.3%)	n/a	
P2: nerves involved, median (IQR)	3.0 (1.0-6.0)	n/a	n/a
P2 nerve count			n/a
1 nerve	4 (33.3%)	n/a	
> 1 nerve	7 (58.3%)	n/a	
Not assessable	1 (8.3%)	n/a	
P3 pattern of invasion			n/a
Intratumoral (IT) only	7 (58.3%)	n/a	
IT + extratumoral (IT&ET)	4 (33.3%)	n/a	
Not assessable	1 (8.3%)	n/a	
P5 intraneural mitosis			n/a
Present	6 (50.0%)	n/a	
Absent	5 (41.7%)	n/a	
Not assessable	1 (8.3%)	n/a	
P6: distance to nearest uninvolved nerve (µm), median (IQR)	94.5 (16.8-250.9)	211.9 (95.6-304.9)	0.264
*Morphological cluster — PNI-positive failures only*			
Morphological cluster (no. of features present)			n/a
0 features	5 (41.7%)	n/a	
1 feature	1 (8.3%)	n/a	
2 features	3 (25.0%)	n/a	
3 features	3 (25.0%)	n/a	
*Adjuvant therapy received*			
Adjuvant therapy received			0.389
None	9 (75.0%)	6 (54.5%)	
RT alone	1 (8.3%)	4 (36.4%)	
RT + CT (CRT)	2 (16.7%)	1 (9.1%)	

Fisher exact (categorical) and Wilcoxon rank-sum (continuous) tests; n/a, not measurable when PNI is absent.

## Discussion

Perineural invasion is increasingly understood not as a passive process of tumour cells tracking along a low-resistance anatomical plane but as an active, invasion-like process driven by reciprocal molecular signalling within a perineural niche of tumour cells, nerves and stroma (3, 8, 9); in oral squamous cell carcinoma (OSCC) the nerve growth factor–TrkA axis is the most consistently implicated mediator and a candidate therapeutic target (10, 11). Against this biological background, the present study examined whether the morphological detail of PNI, rather than its mere presence, carries prognostic information in node-negative OSCC. PNI was identified in 34.0% of the cohort, a figure at the upper end of the 2–82% range reported across squamous carcinoma series (3, 12). This wide range is itself a recognised problem, attributed to the absence of standardised diagnostic criteria and to detection that improves substantially with immunohistochemistry; protein S-100 staining nearly tripled the PNI detection rate on re-review of head and neck specimens in one study (6). Our comparatively high prevalence most plausibly reflects systematic block-level re-review with explicit inclusion of intratumoral foci (which are not always counted in series that emphasise extratumoral nerve involvement), and reinforces the principle that any cross-cohort comparison of PNI is only as reliable as the definition and detection method that underpin it (5).

Most prior series, and the major staging frameworks including the AJCC 8th edition, record PNI only as a binary present-or-absent variable (13). The new CAP protocol (version 4.3.0.0, posted April 2026) mentions reporting the extent and type of PNI and the diameter of the involved nerve in millimetres as optional, emerging elements. In our cohort this binary classification was weakly informative: PNI-positive patients showed a non-significant trend toward inferior disease-free survival (DFS; log-rank p = 0.104) and no difference in locoregional control. A modest single-cohort signal of this kind is concordant with the wider evidence base, given that pooled meta-analysis of 26,062 patients nonetheless demonstrates a robust independent association of PNI with worse overall, disease-free and disease-specific survival (DFS hazard ratio [HR] ≈ 1.7) (14), indicating that the binary effect is genuine but small enough that a 100-patient cohort is underpowered to resolve it. The consensus emerging across the literature, that binary reporting is insufficient and that quantitative PNI parameters should be studied to sharpen prognostic stratification, directly motivated the parameter-level approach taken here.

The single most consistent finding of our analysis was that the calibre of the largest involved nerve provided stronger prognostic differentiation than binary PNI status in this exploratory cohort (noting that binary PNI itself was non-significant here, DFS p = 0.104): an invaded-nerve diameter exceeding 429 µm was associated with an almost fourfold increase in recurrence hazard (HR 3.92, 95% CI 1.18–13.02) and was the most frequently retained variable across 500 bootstrap resamples (64.8%). The prognostic weight of nerve calibre is, however, genuinely contested. Aivazian et al. linked recurrence to involvement of nerves larger than 1 mm.

Beyond calibre, we examined the anatomical pattern, multiplicity and mitotic activity of PNI. An extratumoral component (P3) and involvement of more than one nerve (P2) each yielded point estimates above unity without reaching significance in our small subset, and the literature on these features is openly discordant. For location, the tripartite scheme of Miller et al. and the cohort of Fung et al. found extratumoral PNI to carry the worst prognosis (15, 16), the College of American Pathologists now lists extratumoral extent as an optional reporting element, and a 1,099-patient cohort identified extratumoral PNI as an independent adverse predictor of locoregional control (17); yet Arun et al. and Aivazian et al. found no prognostic difference between location subcategories (18, 19). For multiplicity, Hasmat et al. reported multifocal PNI to be a stronger prognosticator than depth of invasion (20) and Wei et al. showed that stratifying patients by the number of discrete PNI foci (none, one to five, and more than five) separated T1–T2 OSCC into three prognostic groups, with 5-year disease-specific survival of 88.6%, 75.2% and 33.8% respectively (P < 0.001) (21), whereas Miller et al. and Arun et al. found focus number non-prognostic (15, 19). Patterns of recurrence have likewise been shown to differ by PNI configuration in oral tongue cancer (22), and in early-stage disease the location and extent of PNI have been associated with locoregional recurrence (23). Mitotic activity within the PNI focus has been far less studied as an isolated parameter; its inclusion in our score, and in the recent multiparametric system of Nozzoli et al. (24), reflects an emerging interest in proliferative activity at the tumour–nerve interface. The recurring explanation for this discordance (inconsistent methodology, small single-institution cohorts and variable detection) applies equally to our own data and is the central reason these parameters were treated as components of a composite index rather than as standalone predictors.

A specific observation in our cohort was the tendency of three adverse features (large nerve calibre [P1], an extratumoral component [P3] and intraneural mitotic activity [P5]) to cluster together. As reported in the Results, the pairwise correlations among these three parameters were moderate-to-strong and substantially exceeded their correlation with standard clinicopathological variables, and the number of triad features present behaved as a graded ordinal predictor of disease-free survival (HR 1.63 per feature, p = 0.045), with a clear step in event rate between patients carrying none or one feature and those carrying two or three. This pattern suggests that the three features do not occur as independent accidents but co-segregate as a recognisable aggressive PNI phenotype, consistent with the wider observation that extratumoral location tends to accompany worst-pattern-of-invasion type 5 and close surgical margins (17).

These observations motivated the construction of a composite 0–6 morphological score. Importantly, ours is not the first multiparametric PNI score: Nozzoli et al. recently described a six-feature histopathological scoring system, grading nerve number, nerve diameter, nerve depth, intratumoral versus extratumoral location, extent of nerve-sheath involvement and mitotic figures, that stratified head and neck and cutaneous squamous carcinoma into low- and high-risk groups, with the high-risk class showing an approximately fourfold higher recurrence hazard and a near-eightfold higher disease-specific mortality, and was externally validated with 84–96% per-feature concordance (24). Our score shares five of these dimensions but was developed specifically in OSCC and incorporates a sixth, distinct parameter: the minimum distance from the advancing tumour front to the nearest uninvolved nerve (P6). This proximity parameter aligns directly with cancer-neuroscience evidence that nerve–tumour distance, rather than the binary fact of encirclement, governs the nerve injury transcriptome and prognosis, and with the resulting proposal to redefine PNI on a distance basis (11). In our data each additional score point raised the recurrence hazard by 34% (HR 1.34, 95% CI 1.01–1.77, p = 0.044), and the score separated low-risk (score 0–2) from high-risk (score 3–6) patients for both DFS (p = 0.003) and LRC (p = 0.045), with an in-sample concordance index of 0.682. The convergence of an independently derived score with the Nozzoli system, across different anatomical sites and patient populations, is itself reassuring and supports the broader contention that PNI should be reported quantitatively rather than dichotomously.

The relationship between PNI and depth of invasion (DOI) merits specific comment, because the AJCC 8th edition upstages many small tumours to pT3 on DOI alone. PNI and DOI are tightly correlated; in early oral tongue OSCC the median DOI was 4 mm in PNI-negative versus 12 mm in PNI-positive tumours (25); yet Alterio et al. found that in early-stage node-negative disease increasing DOI did not worsen outcome, whereas the absence of PNI conferred a 75% lower relapse probability, making PNI the stronger prognosticator (25). Our bivariable analyses are concordant: adjustment for DOI > 10 mm did not attenuate the P1 effect but increased it (HR 3.96 rising to 6.06), a suppression rather than confounding pattern consistent with the interpretation that DOI partly reflects delayed clinical presentation while PNI more directly indexes aggressive tumour biology.

The clinical corollary of these findings concerns adjuvant radiotherapy. PNI is among the standard adverse features that trigger consideration of postoperative radiotherapy (26, 27), and several authors regard it as an independent prognostic factor that in itself warrants adjuvant treatment, a position reinforced by focused reviews of the role of PNI in treatment decision-making (28, 29). In pathologically node-negative disease Chinn et al. showed PNI to roughly quintuple locoregional failure risk and reported that adjuvant radiotherapy improved locoregional control and disease-free interval in PNI-positive patients (30). Trials confined to isolated PNI, however, have not consistently demonstrated benefit: Liao et al. found no benefit from adjuvant radiotherapy in PNI-positive pT1–3N0 patients (31), and Singareddy et al. similarly found no DFS or locoregional-control advantage in pT1–2N0 oral tongue cancer with isolated PNI (32), while the small irradiated subgroup of Shim et al. was underpowered to detect a difference (33). The prevailing reconciliation is that extent matters: extensive, large-calibre or extratumoral PNI carries the strongest case for radiotherapy, and one group has argued that extratumoral PNI should be reclassified from a minor to a major risk factor (17), whereas focal microscopic PNI as an isolated finding remains a discretionary indication, and lymphovascular invasion may carry broader prognostic weight than PNI across endpoints (34). Within multifactor frameworks PNI functions as one of several minor risk factors that jointly justify postoperative radiotherapy or chemoradiotherapy (35, 36). Our composite score offers a candidate means of operationalising extent: the high-risk band (score 3–6) plausibly identifies the subgroup represented in the positive Chinn-type series, whereas low-score PNI-positive patients may correspond to the negative isolated-PNI cohorts. This hypothesis is explicitly testable, and a recent meta-analysis of postoperative radiotherapy in node-negative early-stage T1/T2 OSCC with isolated perineural invasion likewise emphasises that the available evidence remains insufficient to define a uniform indication (37), supporting the call for prospective randomised evaluation; the score is best regarded as a stratification tool to be evaluated prospectively rather than applied to treatment decisions now.

An apparent paradox in the cohort merits explicit comment: PNI-positive patients did not receive disproportionately more adjuvant therapy than PNI-negative patients. In **Table 1**, surgery alone accounted for 27 of 34 PNI-positive patients (79.4%) versus 54 of 66 PNI-negative patients (81.8%); adjuvant radiotherapy alone was delivered to 4 of 34 PNI-positive patients (11.8%) versus 11 of 66 PNI-negative patients (16.7%); and concurrent chemoradiotherapy was delivered to 3 of 34 PNI-positive patients (8.8%) versus 1 of 66 PNI-negative patients (1.5%). Any adjuvant therapy was delivered to 7 of 34 PNI-positive patients (20.6%) versus 12 of 66 PNI-negative patients (18.2%); this small numerical excess in the PNI-positive group did not approach statistical significance (Fisher exact p = 0.206). The pattern is counterintuitive because PNI is a recognised adverse feature for which postoperative radiotherapy is routinely recommended. Several explanations are likely contributory in this single-centre, real-world cohort: a substantial proportion of PNI-positive patients were T1/T2 lesions with isolated PNI in whom guideline recommendations for adjuvant therapy are themselves debated; multidisciplinary tumour board decisions integrated several adverse features rather than PNI status in isolation; and individual patient factors such as performance status, comorbidities, distance from the treatment centre and patient preference influenced acceptance of adjuvant therapy. This treatment distribution underscores the unmet need for a quantitative morphological score that can identify the PNI-positive subset for whom adjuvant therapy intensification is most likely to be of benefit, rather than treating all PNI-positive patients uniformly.

## Limitations

Several limitations of the present work should be acknowledged. The study was restricted to pathologically node-negative (pN0) patients treated with curative-intent R0 resection. Within this prognostically favourable subgroup, PNI represents one of the few remaining adverse pathological features capable of driving recurrence; this deliberate restriction was intended to allow the morphological characteristics of PNI to be studied without confounding from other dominant adverse features such as nodal disease or involved surgical margins. The findings therefore cannot be directly extrapolated to node-positive disease, close or involved surgical margins, or advanced-stage OSCC with extranodal extension, settings in which competing adverse pathological features are likely to dominate the prognostic landscape. The cohort is single-centre and retrospective, comprising 100 evaluable patients, of whom only 34 were PNI-positive, and the resulting number of recurrence events constrains the precision of every effect estimate; the wide confidence intervals reported throughout reflect this directly. The median potential follow-up of 20.7 months is short relative to comparable head and neck oncology cohorts, and although the numerical separation between the low-risk and high-risk groups defined by our composite score is biologically and statistically encouraging, the long-term durability of this stratification cannot be established, and five-year outcome data — the standard benchmark in OSCC survival analysis — are not yet available; additional recurrence events with longer follow-up may alter the apparent discriminative performance of the score. The morphological cutpoints we propose were derived from this dataset and tested against the same outcome data, so the discriminative performance of the score in our cohort is necessarily an in-sample, optimistic estimate. Interobserver agreement on perineural invasion remained an unresolved problem in the wider literature (38, 39).

A further methodological limitation concerns the assessment of intraneural mitotic activity (P5). Mitotic figure identification on H&E-stained sections remains operator-dependent and is not standardised across centres; in particular, distinguishing genuine intraneural mitoses from those of contiguous infiltrating tumour cells requires careful contextual judgement, and the present count is therefore best regarded as a semi-quantitative estimate. Standardised metrics for quantifying intraneural mitotic activity do not currently exist for routine histopathological practice; future studies would benefit from the use of Ki-67 immunohistochemistry as an objective proliferation index, or from computer-assisted and artificial-intelligence-based tools for nerve detection and comprehensive PNI characterisation, ideally validated against a reproducibility standard, to place this and other PNI parameters on a more quantitatively robust footing before clinical application. As a proof of concept in this direction, a two-stage deep learning prototype for automated PNI detection and characterisation — built on the Phikon-v2 computational pathology foundation model — has been developed by the study group and is available from the corresponding author on reasonable request; in 5-fold cross-validation the prototype achieved AUC-ROC of 0.999 for nerve detection and 0.979 for PNI classification; formal prospective validation against pathologist consensus assessments is ongoing.

## Conclusion

The diameter of the largest involved nerve emerged as the strongest individual predictor of disease-free survival in oral squamous cell carcinoma and the most consistently selected variable in repeated resampling. Three adverse features, namely large nerve calibre, an extratumoral component and intraneural mitotic activity, co-segregated as a recognisable aggressive PNI triad, and a composite 0–6 morphological score significantly stratified PNI-positive patients into low-risk and high-risk groups for both disease-free survival and locoregional control. The principal highlight of this proof-of-concept study is that perineural invasion in OSCC carries a quantifiable morphological spectrum that binary reporting overlooks; these exploratory findings require external prospective validation before the score can be considered in the context of adjuvant treatment strategies.

## Data Availability

The original contributions presented in the study are included in the article/**Supplementary Material**, further inquiries can be directed to the corresponding author/s.

## Supplementary material

Supplementary Figure 1 Kaplan-Meier curves for **(A)** disease-free survival and **(B)** locoregional control, stratified by composite PNI morphological score risk group (Low Risk: score 0–2; High Risk: score 3–6) in the 30 PNI-positive patients with complete morphological data.

Supplementary Figure 2 Missing data heatmap for the analytic cohort (n = 101). Each row represents one patient; each column represents one variable. Missing values are shown in grey.

Supplementary Figure 3 Co-occurrence heatmap of adverse PNI morphological parameter categories. Colour intensity represents the proportion of patients in which both row and column criteria were simultaneously met; values are shown as phi (φ) coefficients.

Supplementary Figure 4 Dot plot of patient-level continuous parameter values (P1: largest nerve diameter; P6: minimum tumour-to-nerve distance) stratified by composite score risk group.

Supplementary Figure 5 Event rate lollipop chart showing DFS and LRC event rates for each PNI morphological parameter category.

Supplementary Figure 6 Bubble plot matrix showing pairwise associations between PNI morphological parameters. Bubble size indicates frequency; colour indicates risk group.

Supplementary Figure 7 Strip chart showing distribution of individual composite PNI scores (0–6) by risk stratification group (Low Risk vs High Risk), with jittering applied to reduce overplotting.

Supplementary Figure 8 LASSO regularisation paths for **(A)** disease-free survival and **(B)** locoregional control. Each coloured line represents one PNI morphological parameter; the vertical dashed line indicates the selected lambda (λ) value at one standard error of the minimum cross-validated partial likelihood deviance.

Supplementary Figure 9 Bland-Altman plot for P1 (largest involved nerve diameter) interobserver agreement (n = 38 PNI-positive cases). The solid red line indicates the mean bias; dashed blue lines indicate the 95% limits of agreement (LoA). Bias = +84.5 µm; 95% LoA: −385 to +554 µm.

Supplementary Figure 10 Bland-Altman plot for P6 (minimum tumour-to-nerve distance) interobserver agreement (n = 96 patients). Bias = −51.4 µm; 95% LoA: −355 to +253 µm.

Supplementary Figure 11 Illustration of P1 (largest nerve diameter) measurement variability between two independent observers annotating the same nerve. Observer 1 (red dashed line): 223.063 µm; Observer 2 (yellow dashed line): 231.631 µm. Subtle differences in the angle and direction of the digital annotation line account for the measurement discrepancy.

Supplementary Figure 12 Example of P6 (minimum tumour-to-nerve distance) measurement variability arising from different location selection at the advancing tumour front. Observer 1 (red): 178.506 µm; Observer 2 (yellow): 163.068 µm. Both measurements are from the same field.

Supplementary Figure 13 Further example of P6 measurement variability when the infiltrating tumour front spans a wide field. Observer 1 (red): 182.645 µm; Observer 2 (yellow): 264.69 µm. In this study, the most-advancing edge was used as the reference point; consensus values were established following joint review of each observer’s independently chosen annotation position.
