# Supplementary material for: Beyond binary: a novel anatomical-geographical-biological framework for perineural invasion risk stratification in oral squamous cell carcinoma – a proof-of-concept study
Source: Front Oncol. 2026 Oct 12;16:1897905. doi: 10.3389/fonc.2026.1897905 (PMC13696292; doi:10.3389/fonc.2026.1897905)
Supplement: Supplementary file 1 [file Supplementaryfile1.pdf]

Supplementary Material

Perineural Invasion in Oral Squamous Cell Carcinoma: A Histopathological Scoring System

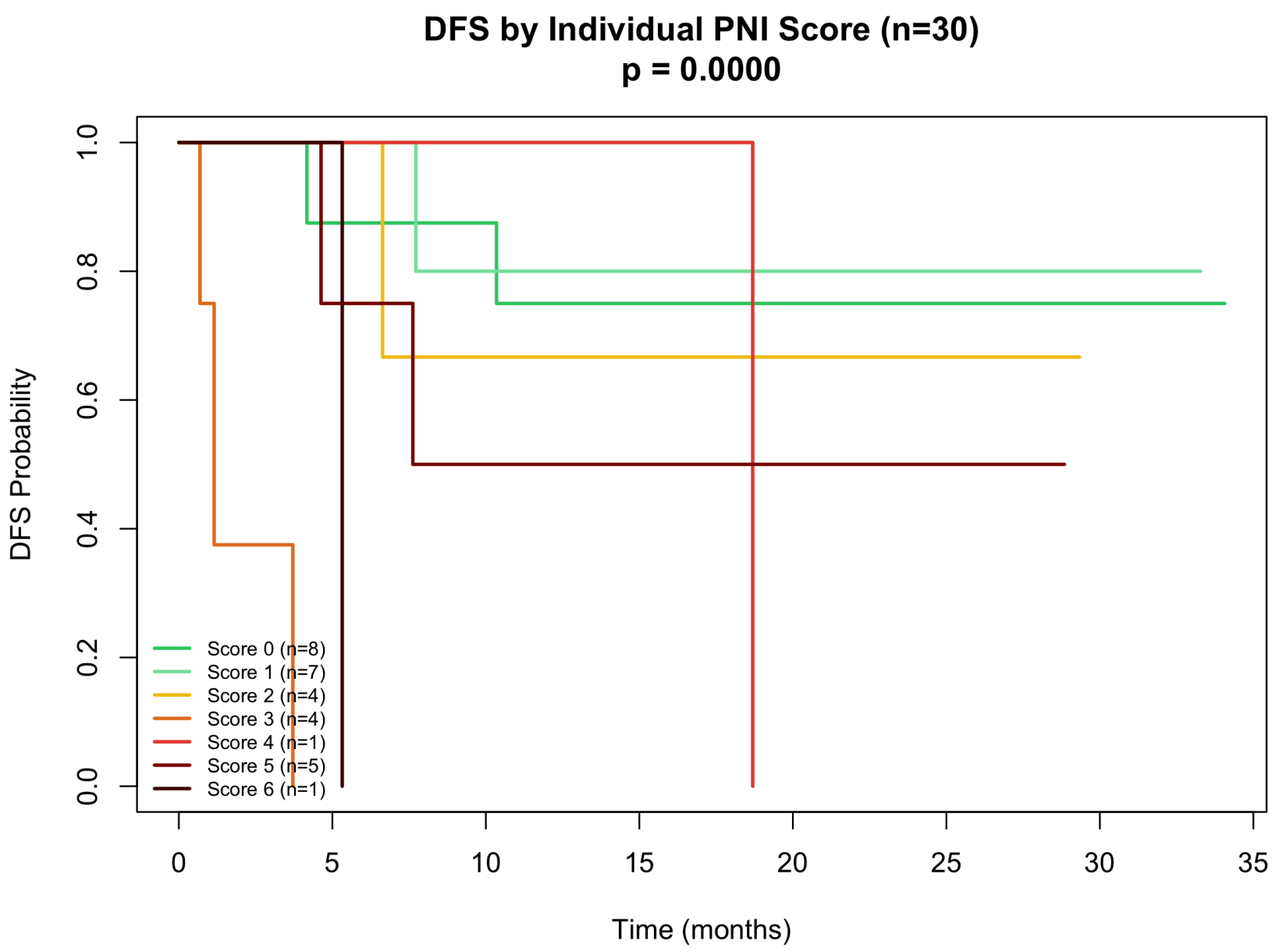

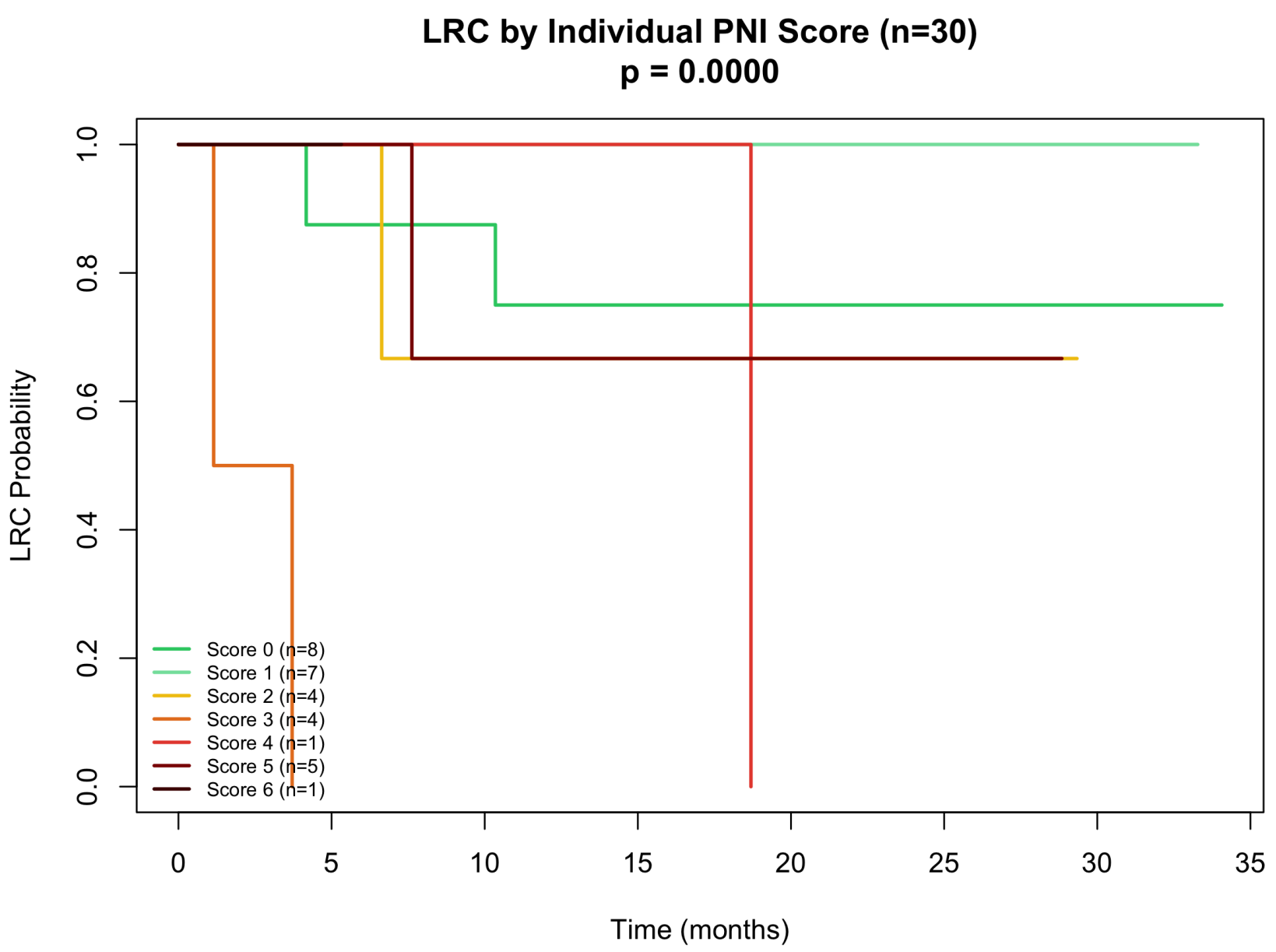

Supplementary Figure 1. Kaplan-Meier curves for (**A**) disease-free survival and (**B**) locoregional control, stratified by composite PNI morphological score risk group (Low Risk: score 0–2; High Risk: score 3–6) in the 30 PNI-positive patients with complete morphological data.

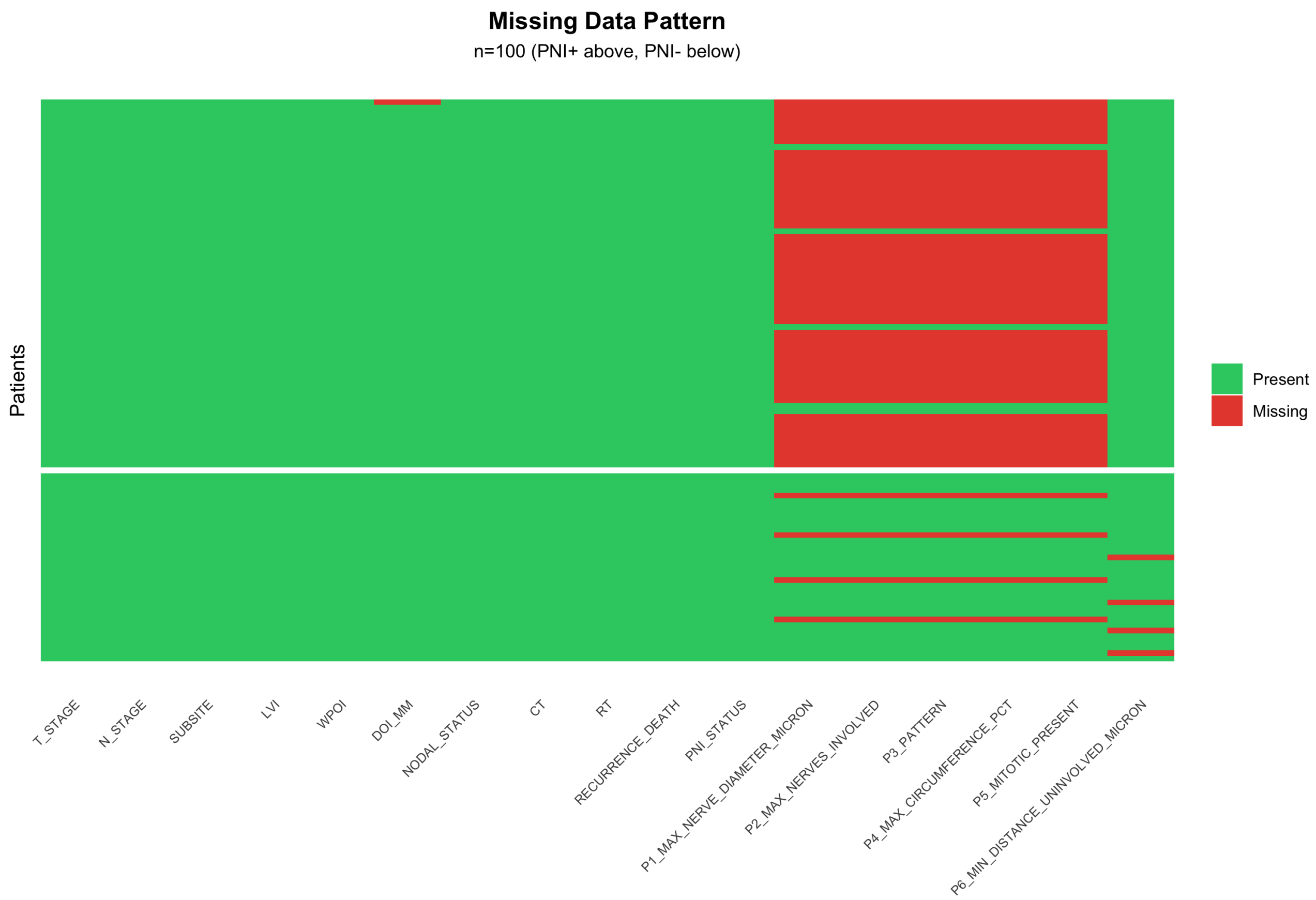

Supplementary Figure 2. Missing data heatmap for the analytic cohort (*n* = 101). Each row represents one patient; each column represents one variable. Missing values are shown in grey.

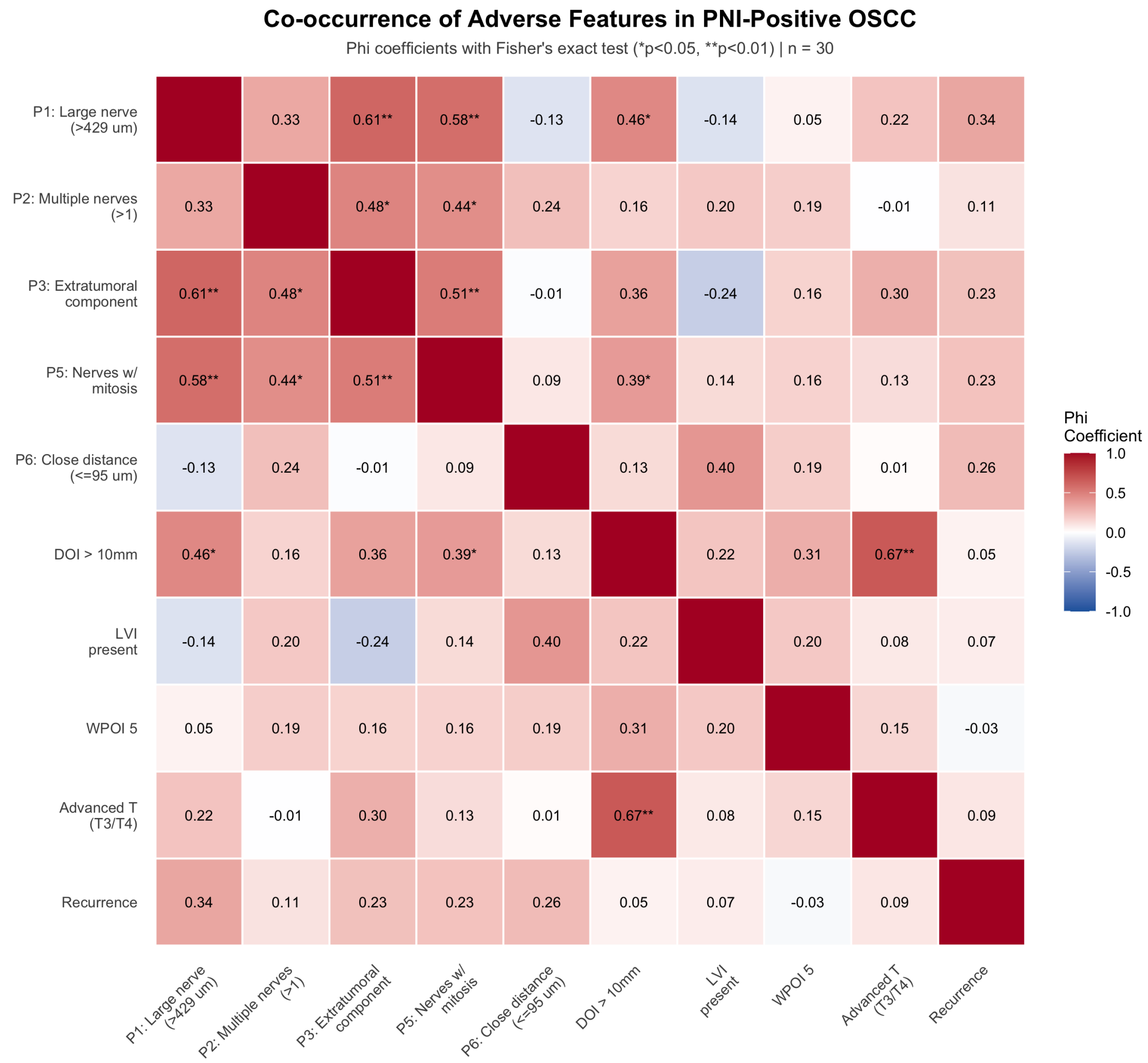

Supplementary Figure 3. Co-occurrence heatmap of adverse PNI morphological parameter categories. Colour intensity represents the proportion of patients in which both row and column criteria were simultaneously met; values are shown as phi (ϕ) coefficients.

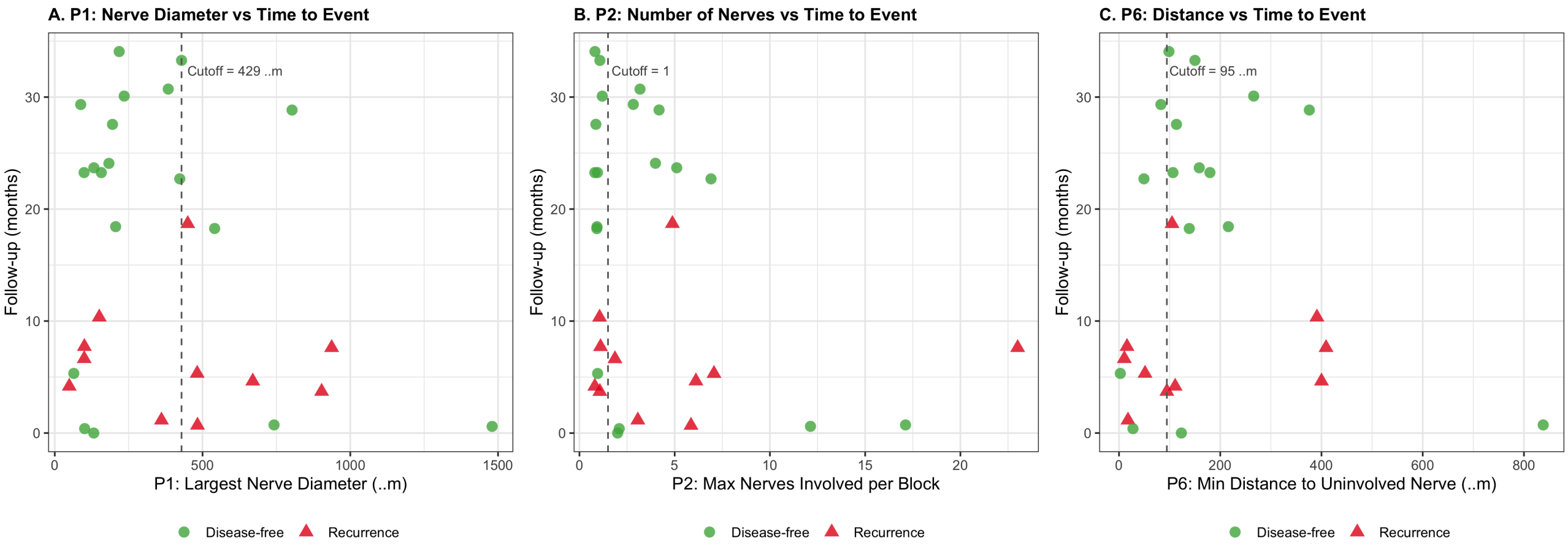

Supplementary Figure 4. Dot plot of patient-level continuous parameter values (P1: largest nerve diameter; P6: minimum tumour-to-nerve distance) stratified by composite score risk group.

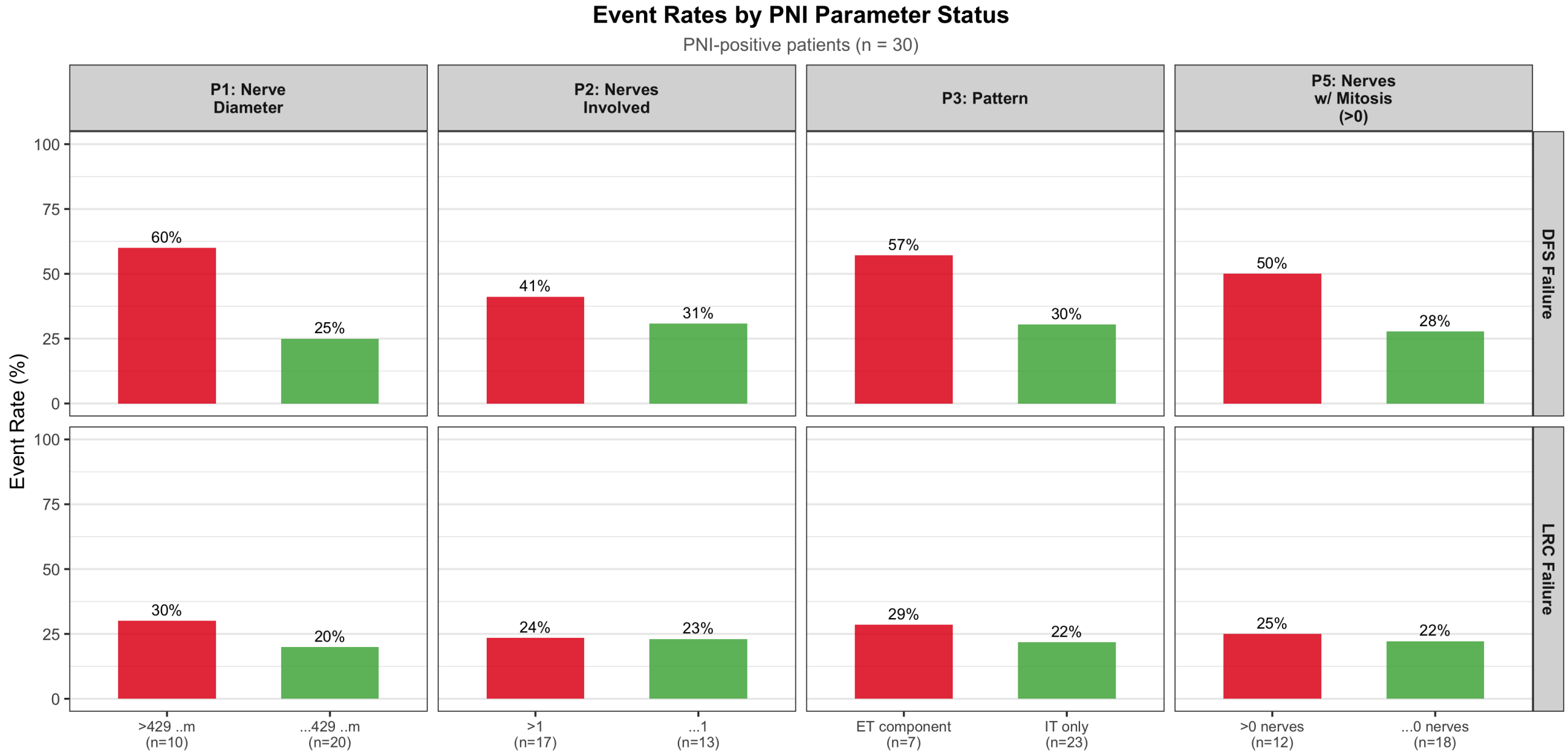

Supplementary Figure 5. Event rate lollipop chart showing DFS and LRC event rates for each PNI morphological parameter category.

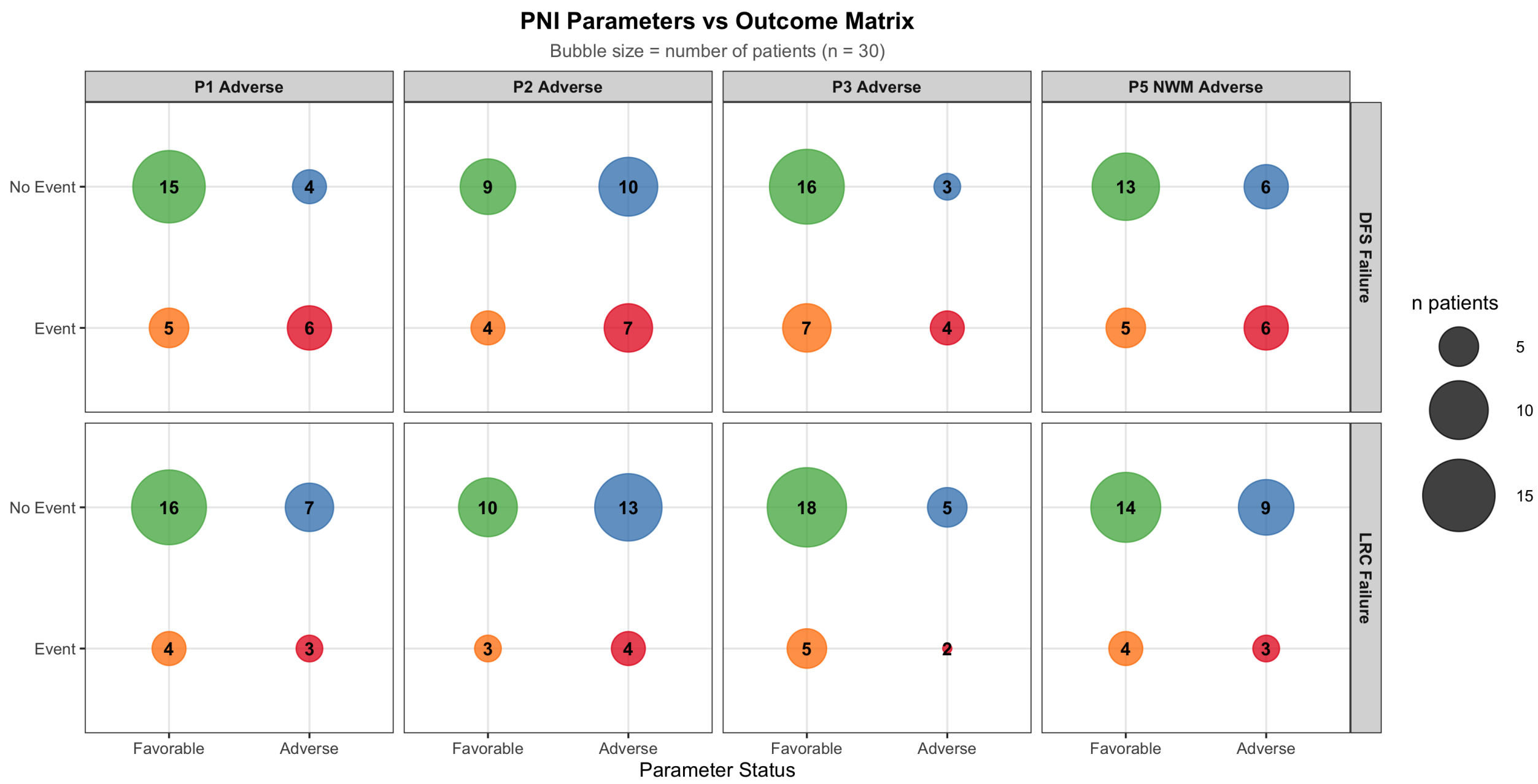

Supplementary Figure 6. Bubble plot matrix showing pairwise associations between PNI morphological parameters. Bubble size indicates frequency; colour indicates risk group.

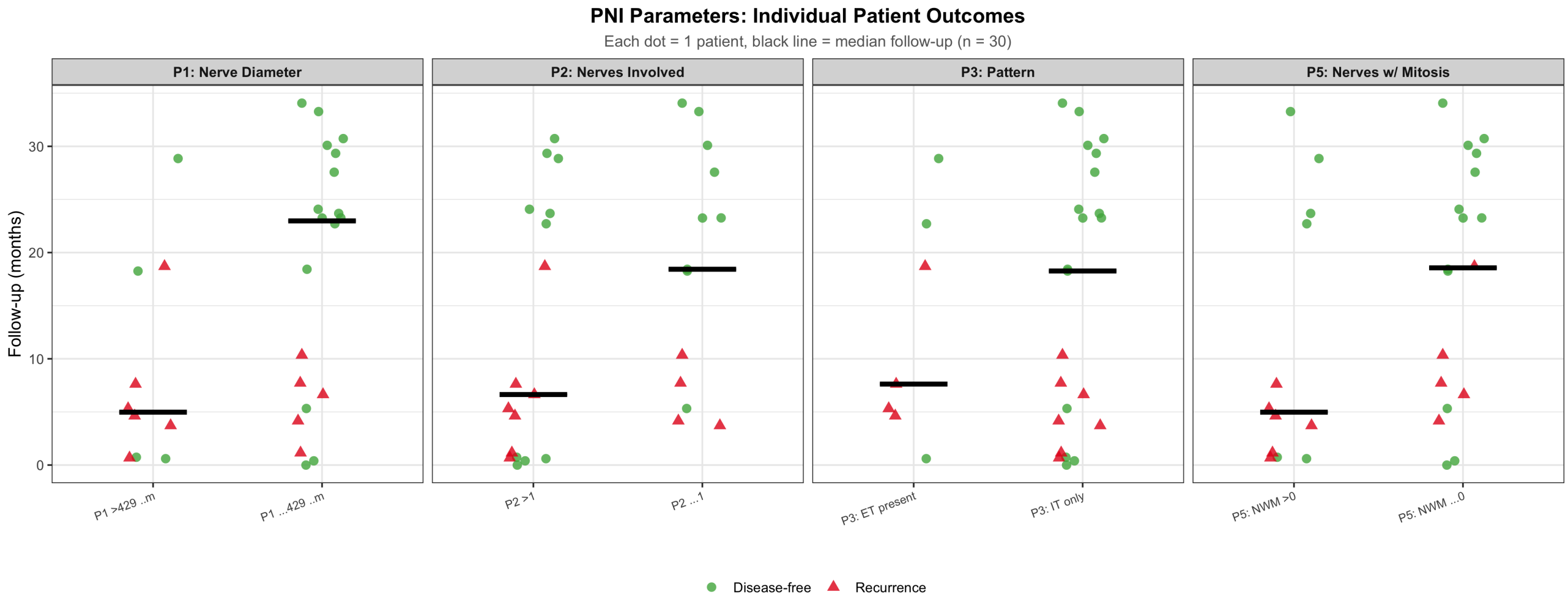

Supplementary Figure 7. Strip chart showing distribution of individual composite PNI scores (0–6) by risk stratification group (Low Risk vs High Risk), with jittering applied to reduce overplotting.

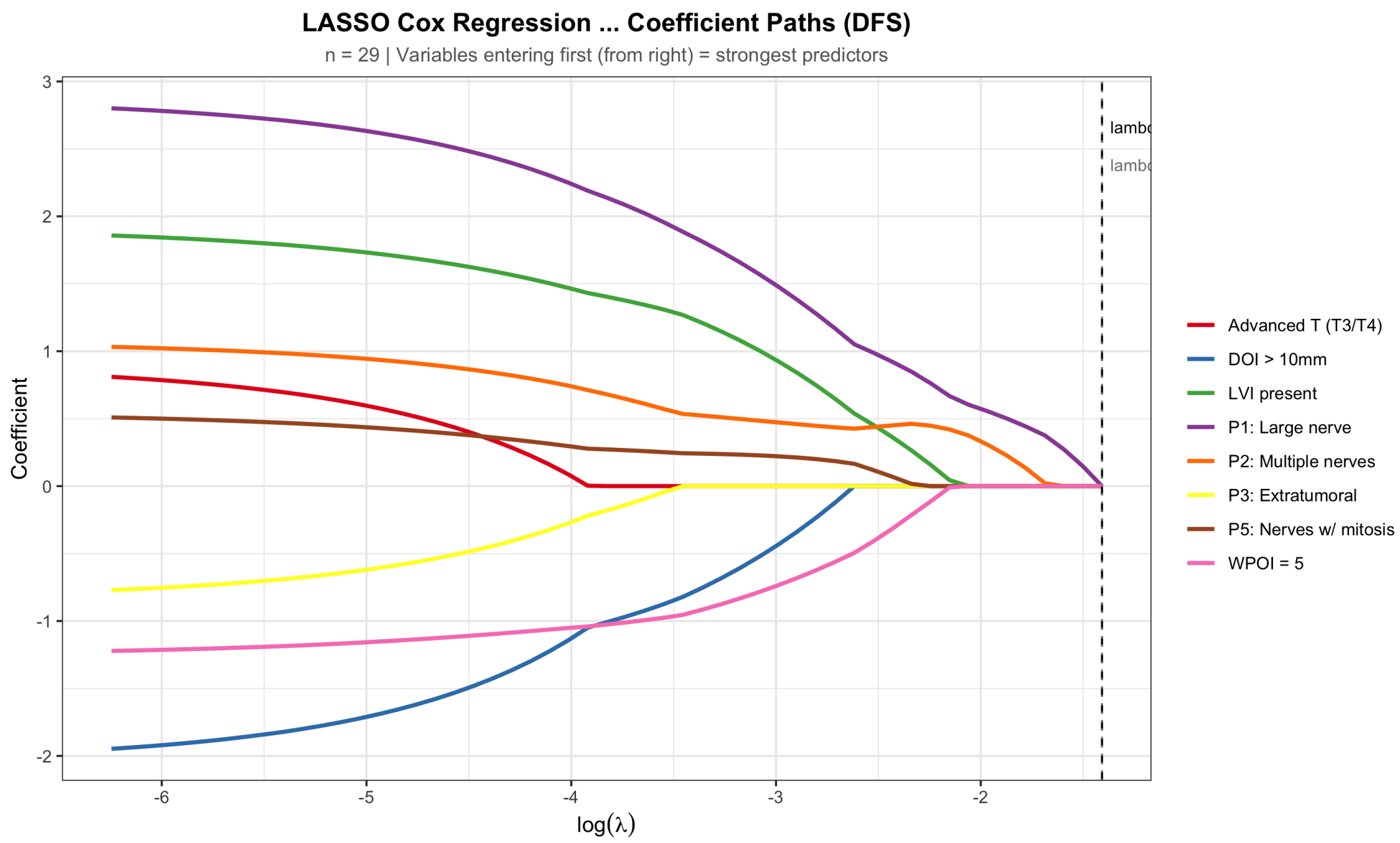

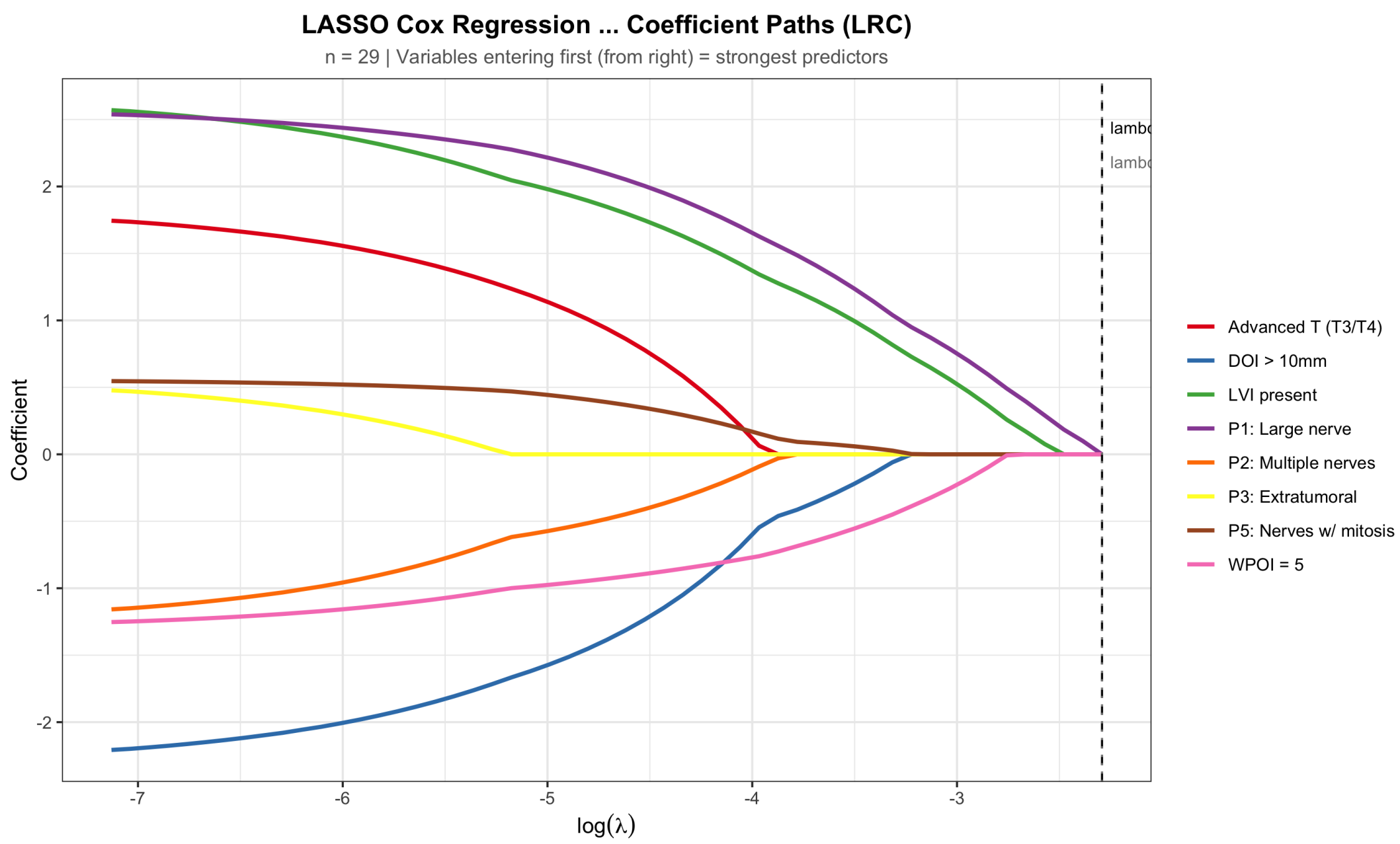

Supplementary Figure 8. LASSO regularisation paths for (**A**) disease-free survival and (**B**) locoregional control. Each coloured line represents one PNI morphological parameter; the vertical dashed line indicates the selected lambda (λ) value at one standard error of the minimum cross-validated partial likelihood deviance.

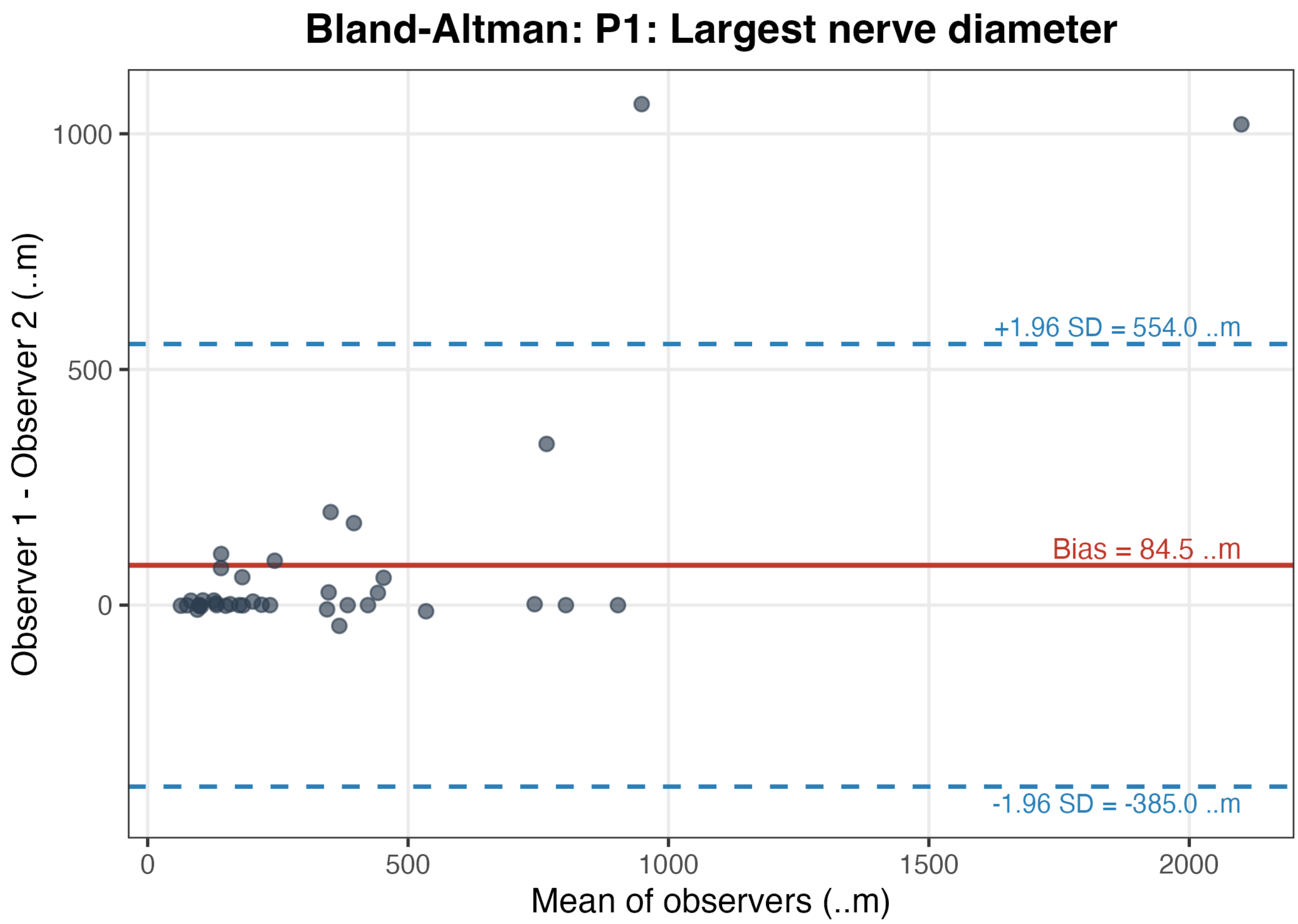

Supplementary Figure 9. Bland-Altman plot for P1 (largest involved nerve diameter) interobserver agreement (*n* = 38 PNI-positive cases). The solid red line indicates the mean bias; dashed blue lines indicate the 95% limits of agreement (LoA). Bias = +84.5 µm; 95% LoA: –385 to +554 µm.

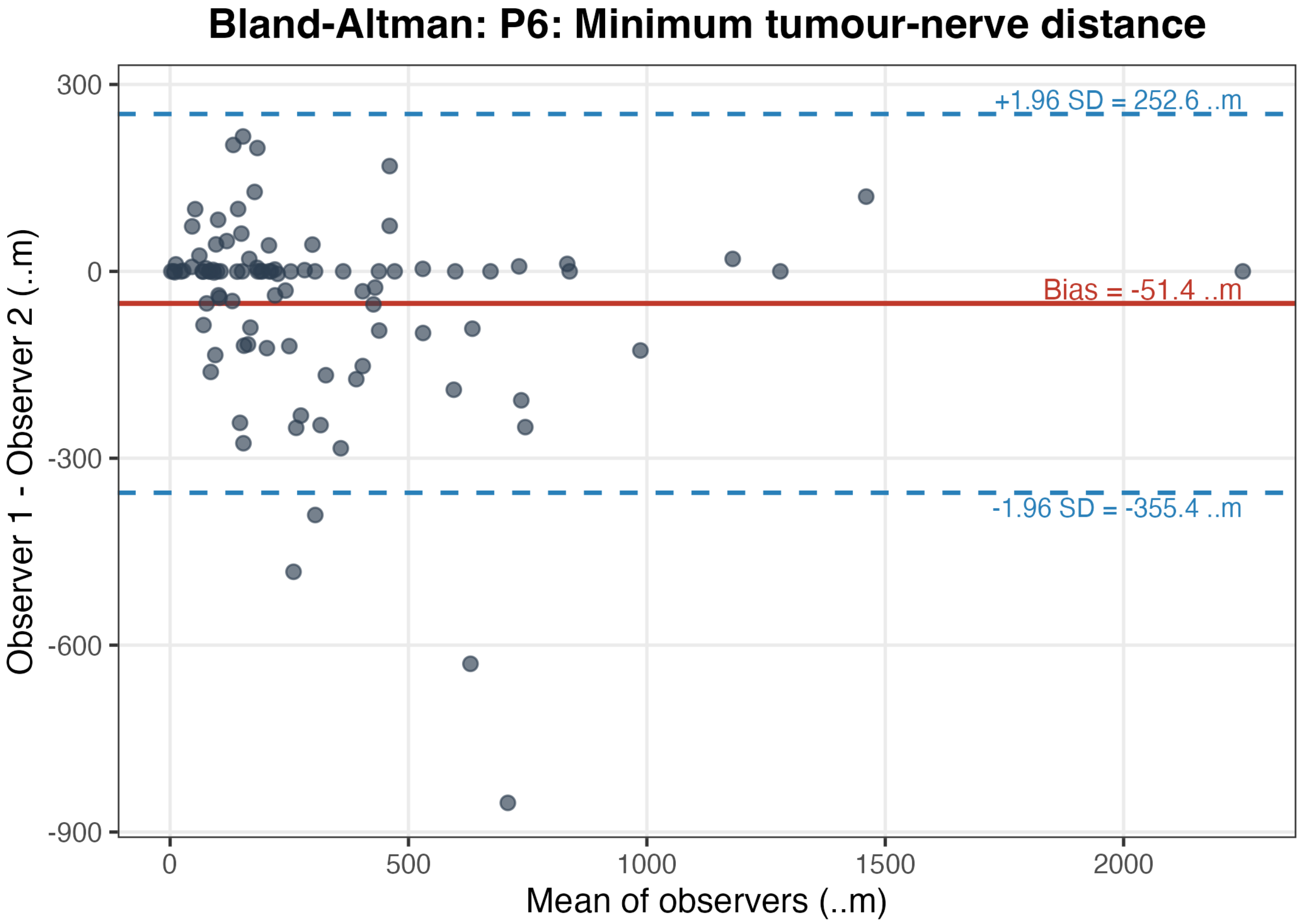

Supplementary Figure 10. Bland-Altman plot for P6 (minimum tumour-to-nerve distance) interobserver agreement (*n* = 96 patients). Bias = –51.4 µm; 95% LoA: –355 to +253 µm.

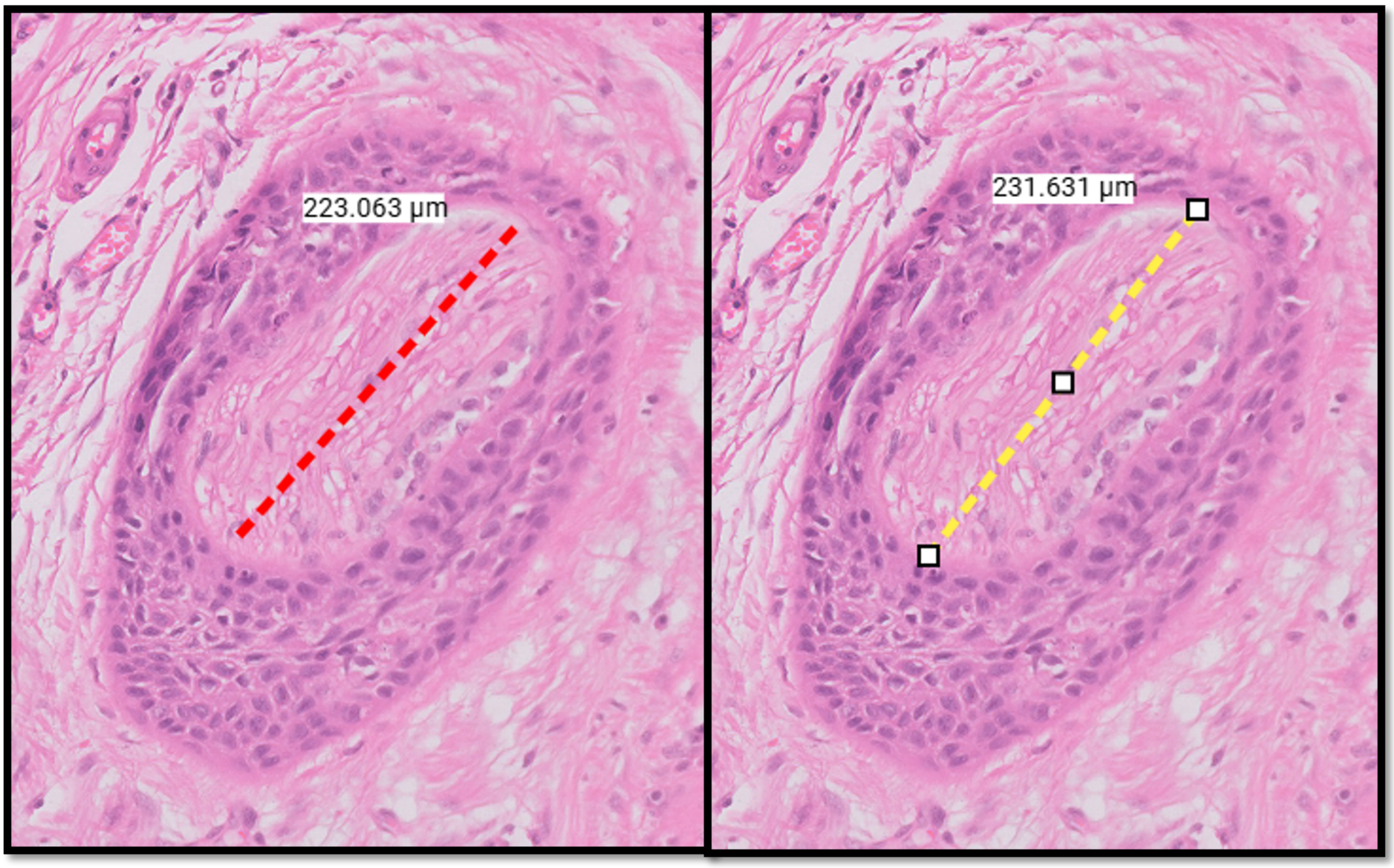

Supplementary Figure 11. Illustration of P1 (largest nerve diameter) measurement variability between two independent observers annotating the same nerve. Observer 1 (red dashed line): 223.063 µm; Observer 2 (yellow dashed line): 231.631 µm. Subtle differences in the angle and direction of the digital annotation line account for the measurement discrepancy.

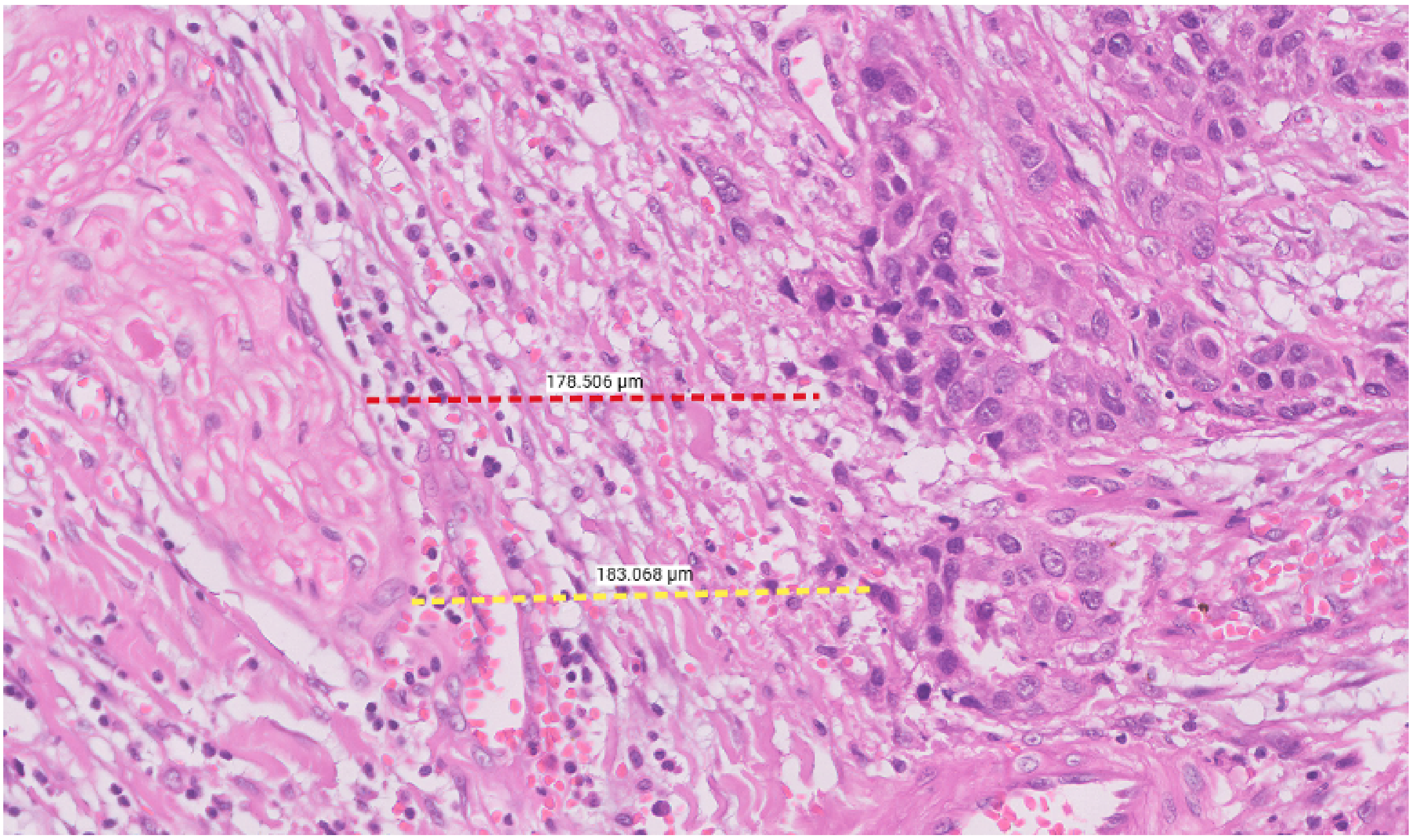

Supplementary Figure 12. Example of P6 (minimum tumour-to-nerve distance) measurement variability arising from different location selection at the advancing tumour front. Observer 1 (red): 178.506 µm; Observer 2 (yellow): 163.068 µm. Both measurements are from the same field.

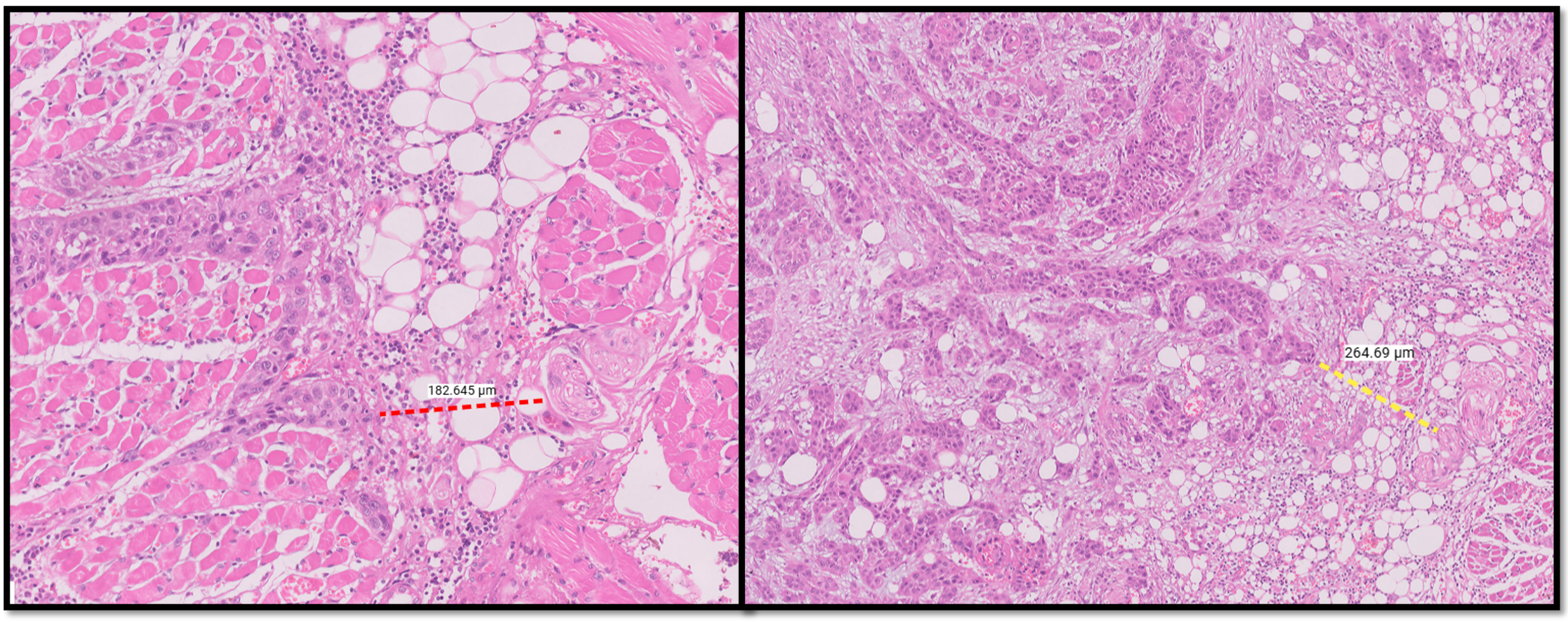

Supplementary Figure 13. Further example of P6 measurement variability when the infiltrating tumour front spans a wide field. Observer 1 (red): 182.645 µm; Observer 2 (yellow): 264.69 µm. In this study, the most-advancing edge was used as the reference point; consensus values were established following joint review of each observer's independently chosen annotation position.
